# High Cure Rates for Hepatitis C Virus Genotype 6 in Advanced Liver Fibrosis With 12 Weeks Sofosbuvir and Daclatasvir: The Vietnam SEARCH Study

**DOI:** 10.1093/ofid/ofab267

**Published:** 2021-06-09

**Authors:** Barnaby Flower, Leanne McCabe, Chau Le Ngoc, Hung Le Manh, Phuong Le Thanh, Thuan Dang Trong, Thu Vo Thi, Hang Vu Thi Kim, Thanh Nguyen Tat, Dao Phan Thi Hong, An Nguyen Thi Chau, Tan Dinh Thi, Nga Tran Thi Tuyet, Joel Tarning, Cherry Kingsley, Evelyne Kestelyn, Sarah L Pett, Guy Thwaites, Vinh Chau Nguyen Van, David Smith, Eleanor Barnes, M Azim Ansari, Hugo Turner, Motiur Rahman, Ann Sarah Walker, Jeremy Day, Graham S Cooke

**Affiliations:** 1 Oxford University Clinical Research Unit (OUCRU), Ho Chi Minh City, Vietnam; 2 Department of Infectious Disease, Imperial College London, United Kingdom; 3 MRC Clinical Trials Unit at UCL, University College London, United Kingdom; 4 Hospital for Tropical Diseases, Ho Chi Minh City, Vietnam; 5 Mahidol-Oxford Tropical Medicine Research Unit (MORU), Mahidol University, Bangkok, Thailand; 6 University of Oxford, United Kingdom; 7 MRC Centre for Global Infectious Disease Analysis, School of Public Health, Imperial College London, United Kingdom

**Keywords:** cirrhosis, direct-acting antivirals, genotype 6, hepatitis C, response-guided therapy

## Abstract

**Background:**

Genotype 6 is the most genetically diverse lineage of hepatitis C virus, and it predominates in Vietnam. It can be treated with sofosbuvir with daclatasvir (SOF/DCV), the least expensive treatment combination globally. In regional guidelines, longer treatment durations of SOF/DCV (24 weeks) are recommended for cirrhotic individuals, compared with other pangenotypic regimens (12 weeks), based on sparse data. Early on-treatment virological response may offer means of reducing length and cost of therapy in patients with liver fibrosis.

**Methods:**

In this prospective trial in Vietnam, genotype 6-infected adults with advanced liver fibrosis or compensated cirrhosis were treated with SOF/DCV. Day 14 viral load was used to guide duration of therapy: participants with viral load <500 IU/mL at day 14 were treated with 12 weeks of SOF/DCV and those ≥500 IU/mL received 24 weeks. Primary endpoint was sustained virological response (SVR).

**Results:**

Of 41 individuals with advanced fibrosis or compensated cirrhosis who commenced treatment, 51% had genotype 6a and 34% had 6e. The remainder had 6h, 6k, 6l, or 6o. One hundred percent had viral load <500 IU/mL by day 14, meaning that all received 12 weeks of SOF/DCV. One hundred percent achieved SVR12 despite a high frequency of putative NS5A inhibitor resistance-associated substitutions at baseline.

**Conclusions:**

Prescribing 12 weeks of SOF/DCV results in excellent cure rates in this population. These data support the removal of costly genotyping in countries where genotype 3 prevalence is <5%, in keeping with World Health Organization guidelines. NS5A resistance-associated mutations in isolation do not affect efficacy of SOF/DCV therapy. Wider evaluation of response-guided therapy is warranted.

## Teaser

Day 14 VL was used to determine whether HCV genotype 6-infected individuals with liver fibrosis should receive 12 or 24 weeks of sofosbuvir and daclatasvir. 100% had low viral load so all were treated for 12 weeks; 100% were cured.

The direct-acting antiviral (DAA) combination of sofosbuvir with daclatasvir (SOF/DCV) is 1 of just 3 pangenotypic regimens recommended by the World Health Organization (WHO) for first-line treatment of adults with chronic hepatitis C virus (HCV) [1]. As the lowest priced option, with the most generic manufacturers worldwide [2], this combination has become the most widely available treatment. Sofosbuvir with daclatasvir can now be procured through voluntary licences in some low- and middle-income countries (LMICs) for as little as US $60 per treatment course [2].

The WHO recommends SOF/DCV for 12 weeks in individuals with mild liver disease, for all HCV genotypes, and for 24 weeks in individuals with liver cirrhosis, with the caveat that 12 weeks “may be considered in countries where genotype distribution is known and Genotype 3 prevalence is <5%” [1]. Although high-quality clinical trial evidence exists for SOF/DCV in genotypes 1–4 [3–8], there are little data on the outcomes of DAA treatment in those with genotype 5 or 6 infection, particularly those with cirrhosis. This is acknowledged in WHO guidelines that advocate for more research into rarer genotypes [1].

Genotype 6 accounts for approximately 5% of global infections but is largely confined to South East Asia [9]. It is a predominant genotype in Vietnam and is responsible for approximately 55% of HCV infections [10]. With 29 confirmed subtypes (6a to 6xf) and 21 unassigned subtypes, it is the most genetically diverse lineage [11], which raises concerns about the potential for naturally occurring resistant variants that may impact treatment outcome [12].

Observational real-world studies suggest that rates of cure of genotype 6 infection with SOF/DCV are high (>90%) [13–17] but may be lower than for genotype 1 or genotype 2 infection, particularly in individuals with cirrhosis.[14, 15] Consequently, the Vietnam national guidelines [15] and the Asia Pacific guidelines [18] diverge from the WHO in recommending that ribavirin should be added to the 12-week SOF/DCV regimen in genotype 6-infected individuals with cirrhosis, or, where ribavirin is contraindicated, 24 weeks of SOF/DCV should be given. This is in contrast to sofosbuvir/velpatasvir (SOF/VEL), the other WHO-approved DAA regimen available through voluntary licences in LMICs, which is recommended for 12 weeks only, without ribavirin, in all guidelines [1, 15, 18].

## Response-Guided Therapy

Although some patients experience virological failure despite 24 weeks of DAA therapy, others can be cured with much shorter durations [19]. In general, baseline characteristics do not predict well those cured with shorter treatment [20], but one hypothesis is that individuals who show a rapid fall in HCV viral load on therapy may be more likely to achieve SVR. Such response-guided therapy (RGT) was routinely used in the era of interferon-based treatment, where an undetectable HCV ribonucleic acid (RNA) at 4 weeks would determine a shortened course of interferon. There is limited evidence to support RGT in the era of DAA therapy [20].

The Vietnam SEARCH study addresses whether individuals with genotype 6 HCV-related liver fibrosis, who exhibit an effective on-treatment virological response, can achieve high rates of cure with 12 weeks of SOF/DCV without need for ribavirin.

## METHODS

### Study Population

Participants were recruited from the outpatient clinic of the Hospital for Tropical Diseases (HTD) in Ho Chi Minh City, Vietnam between February 2019 and June 2020. Eligible patients were 18 years or older, had chronic infection with HCV genotype 6, and severe fibrosis or compensated cirrhosis. Severe fibrosis was defined as a fibroscan score ≥10.1 kPa, which has been shown to correlate to histopathological METAVIR score ≥F3 [21], or a biopsy result consistent with cirrhosis (Ishak score ≥5/6 or equivalent). Compensation was defined as Child-Pugh score ≤7. Participants were required to be HCV-treatment naive, have a body mass index ≥18 kg/m^2^, a creatinine clearance ≥60 mL/min, and no evidence of human immunodeficiency virus (HIV) or hepatitis B coinfection, or solid organ malignancy in the preceding 5 years.

**Figure 1.**
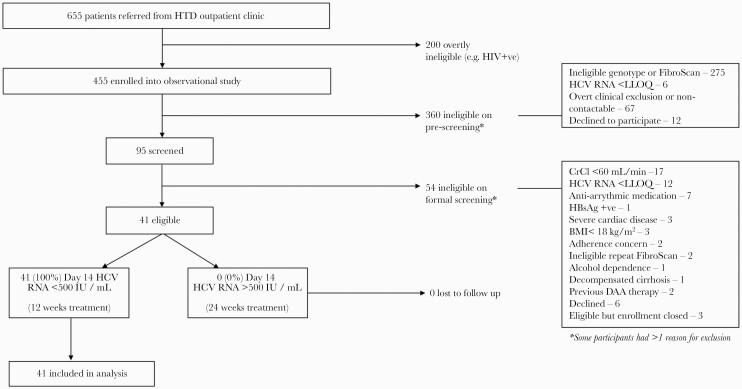
Screening and enrollment. BMI, body mass index; CrCl, creatinine clearance; HBsAg, hepatitis B surface antigen; HCV, hepatitis C virus; HTD, Hospital for Tropical Diseases; LLOQ, lower limit of quantitation; RNA, ribonucleic acid; SD, standard deviation.

Patients referred to the trial were initially enrolled into an observational study, which included fibroscan assessment and genotyping. Eligible individuals from this cohort were invited for additional screening (Figure 1). Full eligibility criteria are provided in the protocol (Online Supplement).

### Study Design

Participants were treated with sofosbuvir 400 mg and daclatasvir 60 mg, administered orally as 2 separate tablets, once daily, with no dose adjustment. Study drugs were procured through Pharco Pharmaceuticals (Alexandria, Egypt).

Hepatitis C virus viral load was measured at baseline and at all subsequent follow-up visits. Day 14 viral load was used to guide duration of therapy: participants with viral load <500 IU/mL at day 14 were treated with 12 weeks of SOF/DCV, and those with HCV RNA ≥500 IU/mL were to continue treatment for 24 weeks. The time point and viral load threshold were chosen on the basis of their use in previous evaluations of response-guided therapy with DAAs [22, 23].

Clinic visits were scheduled on day 0, 14, 28, and monthly thereafter until end of treatment, for 12 or 24 weeks depending on day 14 viral load. End of treatment follow-up visits were scheduled for 4, 8, and 12 weeks after the last tablet for all participants.

The primary endpoint was sustained virological response (SVR12) defined as plasma HCV RNA < lower limit of quantitation (LLOQ) 12 weeks after the end of treatment without prior failure. Failure of first-line treatment during the study was defined as follows: (1) 2 consecutive measurements of HCV RNA > LLOQ (taken at least 1 week apart) after 2 consecutive visits with HCV RNA < LLOQ, at any time during follow up, with the latter confirmatory measurement also being >2000 IU/mL; or (2) 2 consecutive measurements of HCV RNA that are >1 log_10_ above the nadir on treatment and >2000 IU/mL, at any time. Secondary endpoints were lack of initial virological response, serious adverse events (AEs), grade 3/4 clinical AEs, AEs of any grade leading to change in treatment, and adverse reactions (ARs).

### Sample Size Justification

We set a target cure rate of ≥90%, with an unacceptably low cure rate of 70%. Assuming 90% power and one-sided alpha = 0.05, 37 participants were required to exclude the null hypothesis that cure <90%. Assuming 5% loss to follow up, and that, based on pharmacokinetic modeling [24], 90% would achieve on-treatment response and receive 12 weeks (rather than 24 weeks) of therapy, the final target population was 43 participants.

### Study Assessments

Hepatitis C virus RNA was measured using molecular platforms locally. At start of study (for the first 26 participants enrolled), this was the Abbott Architect (LLOQ 12 IU/mL). This was subsequently replaced by the COBAS AmpliPrep/COBAS TaqMan HCV Quantitative Test, version 2.0 (Roche Molecular Systems; LLOQ 15 IU/mL). Hepatitis C virus genotype and subtype were determined using NS5B, Core, 5’ *untranslated region* sequencing, according to a method described by Le Ngoc et al [25]. Assessments during treatment included physical examination, standard laboratory testing, and serum HCV RNA. Adverse events were recorded and graded according to a standardized scale [26].

Whole genome sequencing of the target regions of DCV (NS5A) and SOF (NS5B) was performed for all patients at baseline, with a plan to also assess this at time of detection of any treatment failures. For DCV we report the following RAS (according to European Association for the Study of the Liver [EASL] guidelines for genotype 6 [27, 28]): Q24H, F/L28A/I/L/M/T/V, R30E/H/N/S, L31I/M/V, P32A/L/S/Q/R/S, T58A/G/H/N/S, E92T and T93A/H/N/S. For SOF we report S282G/R/T.

Whole genome sequencing of the HCV viral genome was attained using the Illumina MySeq platform as described previously [29, 30]. The sequence reads were aligned with HCV genotype 6 reference sequence (GenBank accession no. Y12083), and the NS5A (sequence position 6212 to 7567) and NS5B (sequence position 7568 to 9340) region was analyzed. Resistance-associated variants present in more than 1% of sequence reads are reported. We analyzed the sequence for clinically relevant, in vitro, primary, and secondary drug resistance mutations.

### Statistical Analysis

Analyses were performed under intention-to-treat; a per-protocol analysis included all participants taking 90%–110% of prescribed treatment. Proportions and 95% confidence intervals (CIs) were estimated from the marginal effects after logistic regression where possible and calculating proportion with binomial exact 97.5% CIs where not. Absolute HCV VL was analyzed using interval regression adjusting for baseline HCV VL. The proportion of participants with undetectable HCV VL at each visit were analyzed using binomial exact 95% CIs. Analyses were performed using Stata version 16.1.

### Ethical Approval

The trial was registered at www.isrctn.com (Clinical Trial Registration ISRCTN17100273). It was approved by the research ethics committees of The Hospital for Tropical Disease [31], Vietnam Ministry of Health [32], Imperial College London [33], and Oxford University Tropical Research Ethics Committee [34]. The study’s conduct and reporting is fully compliant with the World Medical Association’s Declaration of Helsinki on Ethical Principles for Medical Research Involving Human Subjects [35].

### Patient Consent Statement

Written consent was obtained from all participants. Local ethical committees approved the design of the work, which conforms to standards currently applied in Vietnam and the United Kingdom. Names of authorizing bodies are stated in “Ethical Approval” above and listed in the references.

## RESULTS

### Baseline Characteristics

Of 455 patients screened, 41 were enrolled. Recruitment was completed short of the initial target of 43 as a consequence of coronavirus disease 2019-related restrictions in Vietnam from February 2020. Baseline and clinical characteristics are described in detail in Table 1. All participants had severe fibrosis or compensated cirrhosis, with a median fibroscan score of 17.3 kPa. Ninety-five percent had the minimum Child-Pugh score. There was a high prevalence of hypertension (44%) and diabetes (20%). Only 2 participants had a history of alcohol dependence and no participants reported illicit drug use. The most prevalent subtype was 6a (51%), followed by 6e (34%). Subtypes 6h, 6k, 6l, and 6o were also represented in 1 or 2 participants each.

**Table 1.** Baseline Characteristics^a^

Total participants	41	
Age in years	62	(42–72)
Female	29	(71%)
Body mass index in kg/m^2^	25.3	(19.5–36.5)
Subtype		
6a	21	(51%)
6e	14	(34%)
6h	1	(2%)
6k	1	(2%)
6l	2	(5%)
6o	2	(5%)
Median fibroscan result (kPa)	17.3	(10.1–49.6)
Severe fibrosis by AASLD criteria [45] (10.1–12.5kPa)	10	(24%)
Cirrhosis by AASLD criteria (≥12.5kPa)	31	(76%)
Child-Pugh score		
5	39	(95%)
6	1	(2%)
7	1	(2%)
Baseline HCV viral load in IU/mL	1 030 000	(6258–17 516 779)
HCV viral load, log_10_ IU/mL (range)	6.0	(3.8–7.2)
Previous spontaneous clearance of HCV with reinfection	6	(15%)
Past Medical History		
Illicit drug use	0	(0%)
Alcohol dependence (historic; current excluded)	2	(5%)
Diabetes	8	(20%)
Hypertension	18	(44%)
Ischaemic heart disease	1	(2%)
Stroke	1	(2%)
Malignancy	1	(2%)
Smoking (past, current)	1, 8	(2%, 20%)
Depression	0	(0%)
Chronic obstructive pulmonary disease	0	(0%)
Tuberculosis	0	(0%)
Liver and Kidney Function		
ALT in IU/L (range)	61	(19–216)
AST in IU/L (range)	56	(25–200)
ALP in IU/L (range)	91	(64–249)
Albumin µmol/L (range)	41.6	(30.2–47.9)
Bilirubin µmol/L (range)	11.5	(5.6–34.3)
Creatinine clearance mL/min (range)	76.2	(60.0–176.9)
Platelets K/µL (range)	156	(71–282)
INR (range)	1	(1–1.3)

Abbreviations: AASLD, American Association for the Study of Liver Diseases; ALP, alkaline phosphatase; ALT, alanine aminotransferase; AST, aspartate aminotransferase; HCV, hepatitis C virus; INR, international normalized ratio.

^a^N (%) or median (range) presented.

### Treatment Duration, Adherence to Direct-Acting Antiviral Regimen, and Efficacy Outcomes

By day 14, all 41 participants (100%) had HCV viral loads below the threshold of 500 IU/mL (Table 2); therefore, everyone in the study received 12 weeks of treatment. Ninety-eight percent of participants completed the prescribed course of SOF/DCV. One participant missed 3 doses of SOF/DCV in total, but all participants took 90%–110% of prescribed treatment, meaning intention-to-treat and per-protocol analysis populations were identical. Eight participants (20%) missed at least 1 visit. All 41 participants (100% [97.55% CI, 91.4–100]) achieved SVR12.

**Table 2.** Treatment Outcome^a^

Detectable HCV viral load (HCV VL) at day 14	19	(46%)
Median (IQR) HCV VL at day 14 in IU/mL	42	(28, 96)
Below threshold for extended (24 weeks) therapy	41	(100%)
Above threshold for extended (24 weeks) therapy	0	(0%)
Mean (SD) duration of therapy received in days	84	(0.4)
Median weeks from enrollment to last visit (range)	24	(23–25)
Primary Outcome		
Sustained viral response 12 weeks after end of treatment	41	(100%; 91%–100%)
Secondary Endpoints		
Lack of initial virological response	0	(0%; 0%–9%)
Serious adverse events	1	(2%; 0.1%–13%)^b^
Grade 3/4 clinical adverse events	1	(2%; 0.1%–13%)^b^
Nonserious adverse reactions	20	(49%; 33%–64%)^b^
Adverse events or reactions leading to change in study medication	0	(0%; 0–9%)

Abbreviations: HCV, hepatitis C virus; IQR, interquartile range; SD, standard deviation; VL, viral load.

^a^Where not labeled, data are presented as n (%; 97.5% confidence interval).

^b^95% confidence intervals.

### Viral Kinetics

**Figure 2.**
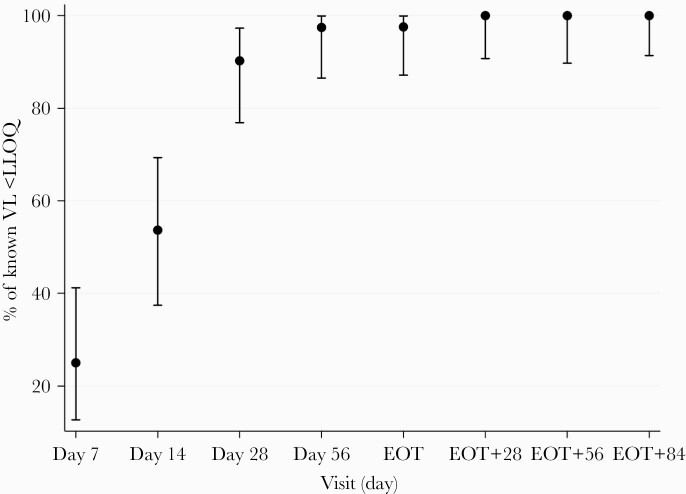
Proportion of participants with hepatitis C virus (HCV) viral load (VL) < lower limit of quantitation (LLOQ). EOT, end of treatment.

**Figure 3.**
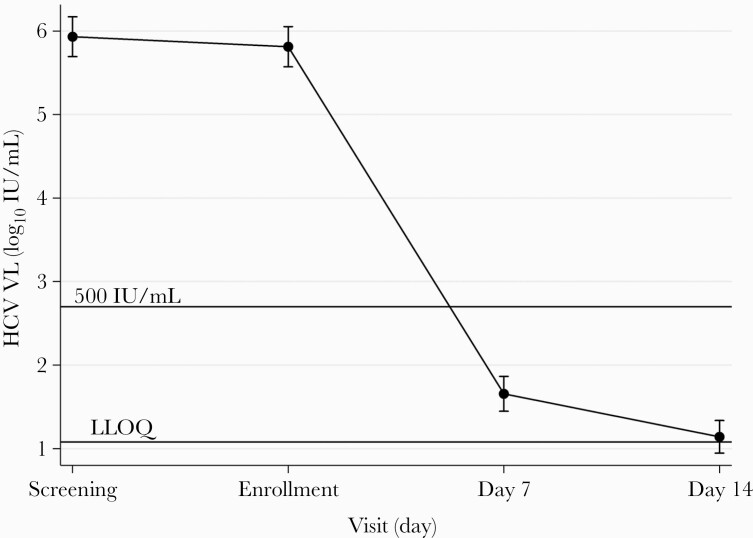
Mean log_10_ hepatitis C virus (HCV) viral load (VL) from day 0 to day 14. Mean log_10_ HCV VL at each visit: Screening 5.93 (95% confidence interval [95% CI], 5.69–6.17); Enrollment 5.81 (95% CI, 5.57–6.05); Day 7 1.75 (95% CI, 1.57–1.93); Day 14 1.20 (95% CI, 1.02–1.38).

Viral clearance was rapid, with no patients showing a lack of initial virological response (97.5% CI, 0%–9%). By day 7, 25% (10 of 40) had HCV RNA <LLOQ, rising to 54% (22 of 41) by day 14, 90% at 4 weeks, 97% at 8 weeks, and 98% at end of treatment (Figure 2). Mean (95% CI) HCV viral load fell from 5.93 log_10_ (5.69–6.17) at baseline to 1.20 (1.02–1.38) at day 14 (Figure 3). At end of treatment, 1 participant had a detectable viral load (HCV RNA 26 IU/mL; Abbott Architect) and 2 had detectable virus <LLOQ. All viral loads measured after end of treatment were undetectable except in 2 individuals: the aforementioned participant with HCV RNA 26 IU/mL at end of treatment, who had detectable HCV RNA < LLOQ at 4 and 12 weeks after end of treatment, and another participant who had undetectable virus at end of treatment but had detectable HCV RNA < LLOQ 8 weeks after end of treatment.

### Viral Genomics

Whole genome viral sequencing of baseline samples revealed 7 distinct resistance-associated substitutions (RAS) to DCV (Table 3). The R30S was detected in 100% (14 of 14) of 6e and the F/L28V RAS were detected in 100% of 6h (1 of 1), 6k (1 of 1), 6l (2 of 2). These RAS almost certainly represent the wild-type (WT) amino acids in these genotypes, but they may still provide some resistance to DCV. The numbers of 6h, 6k, and 6I sequences are low here, but when the available sequences on HCV GLUE [36, 37] are analyzed (8 sequences for 6h, 1 for 6k, and 11 for 6I), F/L28V is present in 100% of sequences.

**Table 3.** Prevalence of Genotype 6 RAS in Cohort (RAS Definitions From EASL Guidelines 2020 [46])

Subtype	n	Daclatasvir RAS Detected (n)	Sofosbuvir RAS Detected (n)
6a	21	F/L28V (3); L31M (1)	L159F^b^ (1)
6e	14	F/L28V (7); L28M (5); R30S (14)^a^; L31I (1); T93S (1)	None detected
6h	1	F/L28V^a^ (1)	None detected
6k	1	F/L28V^a^ (1)	None detected
6l	2	F/L28V^a^ (2)	None detected
6o	2	F/L28V (1); T58A (1) T93S (1)	None detected

Abbreviations: EASL, European Association for the Study of the Liver; RAS, resistance-associated substitutions.

^a^RAS is considered wild type for this genotype.

^b^Not considered clinically relevant RAS for genotype 6 but has been shown to be treatment emergent.

No clinically relevant SOF RAS were detected, but the L159F substitution was detected in one 6a sequence. This substitution has been linked to treatment failure (Table 3). Given that there were no treatment failures, it was not possible to assess the role of these mutations in treatment response. In the 4 participants who exhibited slower virological response, with HCV RNA persisting >LLOQ by day 28, 1 was infected with 6e and 3 had 6a. We detected F/L28V in one of the 6a genomes, but the others had no apparent RAS.

### Safety

Sofosbuvir with daclatasvir was well tolerated in general, and no participants discontinued treatment due to drug side effects. Twenty participants (49%; 95% CI, 33%–64%) reported at least 1 nonserious AR consistent with those described in the summary of product characteristics (SmPC) for SOF/DCV [38]. One participant with type 2 diabetes developed symptomatic hypoglycaemia, which is a known consequence of DAA therapy due to improved glucose control. There was 1 serious AE, a new diagnosis of diffuse large B cell lymphoma, which was diagnosed after discovery of a new splenic mass on an end-of-study ultrasound scan. There were no deaths.

## DISCUSSION

Pangenotypic DAA combinations are not equally efficacious for all HCV genotypes [7, 8] or subtypes [39, 40], and they have lower rates of cure in patients with cirrhosis [1]. This study provides the most detailed dataset to date for the treatment of genotype 6 HCV with SOF/DCV in patients with severe fibrosis or cirrhosis.

Initial virological response was more rapid than expected, with an almost 5-log drop in mean viral load after 7 days of therapy. Previous studies in HCV kinetics have shown a biphasic viral decline in response to DAA therapy, characterized by a rapid 3–4 log viral decline from 12 to 48 hours, followed by a slower second phase from day 2 onwards [41].

The high cure rate observed is consistent with real-world studies [14, 15]. In the largest published cohort of genotype 6-infected individuals treated with SOF/DCV, from Phnom Penh in Cambodia, among 1292 patients with a mixture of mild disease and cirrhosis, 95.9% (95% CI, 94.7–96.9) of patients with a known treatment outcome achieved SVR12 with 12 weeks SOF/DCV (without ribavirin) [14]. A separate analysis from the same study, restricted to patients with compensated cirrhosis (59.7% genotype 6), found a 98.1% cure rate (95% CI, 97.5–98.6]) with this regimen. In Vietnam, 1111 of 1148 (96.8%) individuals with genotype 6 infection treated with either SOF/DCV or SOF/ledipasvir achieved SVR with 12 weeks of therapy [15]. Liver fibrosis did not influence outcome.

The additional evidence from our study is relevant to current treatment guidelines. First, in most LMICs, SOF/DCV is cheaper than SOF/VEL and easier to procure. There are 5 WHO prequalified generic suppliers for SOF and 3 for DCV, contrasting with just 1 supplier of the SOF/VEL combination [2]. Three DCV products have been registered in Vietnam since the fourth quarter of 2019, which may lead to further decline in DCV price in the future [2]. Data showing high cure rates after 12 weeks treatment duration will promote its use.

Second, we demonstrated that high cure rates are possible in patients with liver fibrosis with SOF/DCV in the absence of adjunctive ribavirin, which has a problematic side-effect profile. Ribavirin is teratogenic and causes anaemia, so that patients taking it must be monitored for signs of toxicity. International guidelines recommend nonribavirin-based treatment where possible [1, 27], and our data add to those suggesting its use is unnecessary.

Third, 12 weeks of DAA treatment is cheaper than 24 weeks. In Vietnam, DAAs remain relatively expensive, with a 12-week course of SOF/DCV currently priced at $1347 [2]. When the additional laboratory tests and healthcare visits involved in longer course therapy are factored in, savings are likely to be substantial [2].

Finally, the 2018 WHO treatment guidelines recommend 24 weeks of treatment with SOF/DCV for patients with compensated cirrhosis, but that 12 weeks “may be considered in countries where genotype distribution is known and genotype 3 prevalence is known to be <5%” [1]. This guidance is measured, owing to the limited data for genotypes 5 and 6. Our data for genotype 6 supports this notion and suggests that in Vietnam, where genotype 3 prevalence is approximately 0.5% [10], there is no need for costly genotyping. As well as driving down the price of treatment, this would also help decentralize hepatitis care, ending the reliance on well resourced laboratories that offer genotyping.

In the absence of slow treatment responders or treatment failures, it is not possible to draw conclusions from this study regarding a role for response-guided therapy. However, although the cost of viral load testing (currently $20–$50 in Vietnam) remains well below the cost of drugs, RGT has the potential to provide significant financial savings and appears to be worth pursuing.

In general, RAS have been reported with reference to their significance in other, non-6, HCV genotypes. Their significance in HCV genotype 6 is not well understood, particularly for non-6a subtypes. Six different genotype 6 subtypes were seen in our study (6a, e, h, k, l, o). We found that several polymorphisms previously reported as RAS appear to be WT in some genotype 6 subtypes, and their contribution to resistance and treatment failure remains unclear. It has been shown that certain HCV subtypes (eg, 4r and 3b) are inherently resistant to NS5A inhibitors; in vitro studies suggest that this may also be true for certain genotype 6 subtypes [12]. There was no obvious contribution of RAS to treatment outcomes in this study. However, further clinical studies are needed to elucidate the role of RAS in genotype 6 infections, especially in the context of other predictors of poor treatment response, such as decompensated cirrhosis [20].

We found SOF/DCV to be well tolerated. No patients stopped treatment due to side effects. However, nonsevere ARs typical of those described in SOF/DCV SmPC, such as dizziness and gastritis, were reported by approximately half the study population. One participant had a serious AE: a new splenic mass detected by ultrasound at end of study, ultimately diagnosed as B-cell lymphoma. B-cell non-Hodgkin’s lymphoma is a typical extrahepatic manifestation of chronic HCV, and emergence after HCV eradication with DAA therapy has been described [42]. Three participants had low levels of virus detectable at end of treatment; all achieved SVR12. In clinical practice, routine testing for HCV RNA at end of treatment is not recommended; our experience here supports recent data showing that treatment prolongation is not indicated in this scenario [43, 44].

Our study has some limitations. Stringent eligibility criteria meant that only 6% of patients screened were enrolled, which could affect the generalizability of our results. The participating cohort did not include people who inject drugs or individuals with HIV, hepatitis B coinfection, or renal impairment. These are important groups among the global HCV population. Adherence was very high and may not reflect real-world practice.

Furthermore, only 31 participants had cirrhosis by American Association for the Study of Liver Diseases (AASLD) criteria [45]. We did not include patients with decompensated cirrhosis, and all but 2 of the individuals with cirrhosis had the minimum Child-Pugh score. Treatment outcomes in individuals with decompensated cirrhosis are inferior to those in seen in compensated disease [14, 15]. However, our findings are likely generalizable to a significant proportion of genotype 6-infected individuals with compensated cirrhosis in Asia.

## CONCLUSIONS

In summary, this study shows that excellent outcomes are achievable with 12 weeks treatment of SOF/DCV for patients with HCV genotype 6 infection and compensated cirrhosis. More research is required to assess the utility of early virological response in further shortening therapy and to determine the role of genotype 6-associated nucleoside substitutions in treatment response.

## Supplementary Data

Supplementary materials are available at *Open Forum Infectious Diseases* online. Consisting of data provided by the authors to benefit the reader, the posted materials are not copyedited and are the sole responsibility of the authors, so questions or comments should be addressed to the corresponding author.

ofab267_suppl_Supplementary_Material
