# Supplementary material for: High Cure Rates for Hepatitis C Virus Genotype 6 in Advanced Liver Fibrosis With 12 Weeks Sofosbuvir and Daclatasvir: The Vietnam SEARCH Study
Source: Open Forum Infect Dis. 2021 Jun 9;8(7):ofab267. doi: 10.1093/ofid/ofab267 (PMC8320300; doi:10.1093/ofid/ofab267)
Supplement: ofab267_suppl_Supplementary_Material [file ofab267_suppl_supplementary_material.docx]

| A pilot study to assess shortened therapy for hepatitis C infected adults in Vietnam  Short title: SEARCH-1 - Southeast Asian Research Collaborative in Hepatitis | | |
| --- | --- | --- |
| Version: | 3.3 | |
| Date: | 25^th^ July 2018 | |
|  | | |
| Clinical trial registration #: | | |
|  | |  |
| SponsOR: | | **Imperial College, London** |
|  | |  |
| **FUNDER:** | | **MRC (UK)** |
| **Authorised by:** | | |
| Name: | | Professor Graham Cooke |
| Role: | | Chief Investigator |
| Signature: | |  |
| Date: | |  |
|  | |  |
| Name: | | Professor Ann Sarah Walker |
| Role: | | Lead Trial Statistician |
| Signature: | |  |
| Date: | |  |
| Name:  Role:  Signature:  Date: | | **Evelyne Kestelyn** |
|  |  | **Head of OUCRU CTU** |
|  |  |  |
|  |  |  |
| Name: | | Professor Guy Thwaites |
| Role: | | Director OUCRU |
| Signature: | |  |
| Date: | |  |
| Name: | | **Dr Nguyen Van Vinh Chau** |
| Role: | | **Director, Hospital for Tropical Diseases** |
| Signature: | |  |
| Date: | |  |
| Name: | | **Professor Jeremy Day** |
| Role: | | **Head, CNS and HIV Infections Research Group, OUCRU** |
| Signature: | |  |
| Date:  Name: | | **Professor Ellie Barnes** |
| Role: | | **Professor of Hepatology, University of Oxford** |
| Signature:  Date: | |  |
| Name:  Role: | | **Dr Motiur Rahman**  **Head of Laboratories, OUCRU** |
| Signature:  Date: | |  |
| Name: | | **Dr Hugo Turner** |
| Role: | | **Lead Health Economist, OUCRU** |
| Signature:  Date: | |  |
| Name:  Role: | | **Professor Joel Tarning**  **MORU** |
| Signature:  Date: | |  |

Statement of Compliance

The trial will be conducted in accordance with the ICH E6, the Code of Federal Regulations on the Protection of Human Subjects (45 CFR Part 46), and the Terms of Award. The Chief Investigator will assure that no changes to the protocol will take place without prior agreement from the sponsor and documented approval from the Institutional Review Board (IRB), except where necessary to eliminate an immediate hazard(s) to the trial participants.

Sponsor

Imperial College London is the trial Sponsor and has delegated responsibility for pharmacovigilance, quality assurance and quality control, document management (including the Trial master file), database and archiving, regulatory and ethics approvals to the OUCRU CTU in Ho Chi Minh City, Vietnam. Queries relating to sponsorship of this trial should be addressed to the Imperial Joint Research Compliance Office via the Chief Investigator.

Funding

The trial is funded in by the the Global Challenges Research Fund (GCRF) from the UK Medical Research Council (MRC).

Authorisations and Approvals

This trial was approved by [Insert after REC approval]

Trial Registration

This trial will be registered with the International Standard Randomised Controlled Trial Register (number to be added).

I agree to ensure that all staff members involved in the conduct of this study are informed about their obligations in meeting the above commitments.

Chief Investigator: Prof. Graham Cooke

Signed: Date:

key trial contacts

Coordinating Site

| OUCRU CTU | Tel:  Email: | +84 (0) 83924 1983  ekestelyn@oucru.org |
| --- | --- | --- |

OUCRU Staff

| Trial Manager: | Dang Trong Thuan | Tel:  Email: | +84 (0)989876526  thuandt@oucru.org |
| --- | --- | --- | --- |
| Data Manager: | Ho Van Hien | Tel:  Email: | +84 (0) 988 084 391  hienhv@oucru.org |
| Trial Statistician: | Dr. Ronald Geskus | Tel:  Email: | +84 (0) 911 621 217 rgeskus@oucru.org |
| Clinical Project Manager: | TBC | Tel:  Email: |  |
| Head of CTU: | Evelyne Kestelyn | Tel:  Email: | +84 (0)904316180  [ekestelyn@oucru.org](mailto:ekestelyn@oucru.org) |

Chief Investigator

| Professor Graham Cooke | Tel:  Email: | +447773705678  g.cooke@imperial.ac.uk |
| --- | --- | --- |

Co-investigators

| Professor Guy Thwaites | Director, OUCRU, Ho Chi Minh City, Vietnam |
| --- | --- |
| Dr Nguyen Van Vinh Chau | Director, Hospital for Tropical Diseases, Ho Chi Minh City, Vietnam |
| Professor Jeremy Day | Head, CNS and HIV Infections Research Group, OUCRU, Ho Chi Minh City, Vietnam |
| Dr. Nguyen Thanh Dung | Vice-Director, Hospital for Tropical Diseases, Ho Chi Minh City, Vietnam |
| Dr. Le Manh Hung | Vice-Director, Hospital for Tropical Diseases, Ho Chi Minh City, Vietnam |
| Dr. Le Thanh Phuong | Head of Out-patient department, Hospital for Tropical Diseases, Ho Chi Minh city, Vietnam |
| Dr. Dao Bach Khoa | Head of In-patient department, Hospital for Tropical Diseases, Ho Chi Minh city, Vietnam |
| Professor Ellie Barnes | Professor of Hepatology, University of Oxford |
| Professor Ann Sarah Walker | MRC Clinical Trials Unit at UCL, 90 High Holborn, London, WC1V 6LJ |
| Dr Sarah L Pett | MRC Clinical Trials Unit at UCL, 90 High Holborn, London, WC1V 6LJ |
| Dr Motiur Rahman | Head of Laboratories, OUCRU, Ho Chi Minh City, Vietnam |
| Evelyne Kestelyn | Head of CTU, OUCRU, Ho Chi Minh City, Vietnam |
| Professor Joel Tarning | Head of Clinical Pharmacology, Mahidol Oxford Tropical Medicine Resarch Unit, Bangkok, Thailand |

table of CONTENTS

[Statement of Compliance 3](#_Toc520289208)

[key trial contacts 4](#_Toc520289209)

[table of CONTENTS 6](#_Toc520289210)

[Summary of Trial 9](#_Toc520289211)

[Trial Schema 10](#_Toc520289212)

[TRIAL ASSESSMENT SCHEDULE 11](#_Toc520289213)

[Abbreviations 12](#_Toc520289214)

[1 Background information and scientific rationale 15](#_Toc520289215)

[2 trial design and endpoints 20](#_Toc520289216)

[2.1 Description of trial design 20](#_Toc520289217)

[2.2 trial endpoints 20](#_Toc520289218)

[3 Participant identification 21](#_Toc520289219)

[3.1 Inclusion Criteria 21](#_Toc520289220)

[3.2 Exclusion Criteria 22](#_Toc520289221)

[3.3 Number of Participants 23](#_Toc520289222)

[4 REGISTRATION AND RECRUITMENT 24](#_Toc520289223)

[4.1 Recruitment and retention strategies 24](#_Toc520289224)

[4.2 Screening Procedures & Pre-enrolment Investigations 24](#_Toc520289225)

[4.3 informed consent 24](#_Toc520289226)

[4.4 Co-enrolment Guidelines 25](#_Toc520289227)

[5 TReatment of PARTICIPANTS 26](#_Toc520289228)

[5.1 Treatment Schedule 26](#_Toc520289229)

[5.2 Protocol Treatment Discontinuation 27](#_Toc520289230)

[6 Assessments & Follow-up 28](#_Toc520289231)

[6.1 Trial Assessment Schedule 28](#_Toc520289232)

[6.2 Procedures for Assessing Efficacy 28](#_Toc520289233)

[6.3 Procedures for Assessing Safety 28](#_Toc520289234)

[6.4 Other assessments 29](#_Toc520289235)

[6.5 Early Stopping of Follow-up 30](#_Toc520289236)

[6.6 Loss to Follow-up 31](#_Toc520289237)

[6.7 Completion of protocol follow up 31](#_Toc520289238)

[7 investigational medicinal product 32](#_Toc520289239)

[7.1 Introduction 32](#_Toc520289240)

[7.2 SOFOSBUVIR (SOF) 32](#_Toc520289241)

[**7.2.1** **PRODUCT** 32](#_Toc520289242)

[**7.2.2** **TREATMENT SCHEDULE** 33](#_Toc520289243)

[**7.2.3** **DISPENSING** 33](#_Toc520289244)

[**7.2.4** **DRUG-DRUG INTERACTIONS** 33](#_Toc520289245)

[**7.2.5** **DOSE MODIFICATIONS, INTERRUPTIONS & DISCONTINUATIONS** 34](#_Toc520289246)

[7.3 DACLATASVIR (DCV) 34](#_Toc520289247)

[**7.3.1** **PRODUCT** 34](#_Toc520289248)

[**7.3.2** **TREATMENT SCHEDULE** 34](#_Toc520289249)

[**7.3.3** **DISPENSING** 34](#_Toc520289250)

[**7.3.4** **DRUG-DRUG INTERACTIONS** 35](#_Toc520289251)

[**7.3.5** **Dose Modifications, Interruptions & Discontinuations** 35](#_Toc520289252)

[7.4 Blinding 36](#_Toc520289253)

[7.5 Overdose of Trial Medication 36](#_Toc520289254)

[7.6 Protocol Treatment Discontinuation 36](#_Toc520289255)

[7.7 Adherence 37](#_Toc520289256)

[7.8 Treatment Data Collection 37](#_Toc520289257)

[7.9 Non-trial Treatment 37](#_Toc520289258)

[**7.9.1** **Treatment After end of study drug** 37](#_Toc520289259)

[8 Safety Reporting 38](#_Toc520289260)

[8.1 Definitions 38](#_Toc520289261)

[**8.1.1** **Medicinal Products** 39](#_Toc520289262)

[**8.1.2** **Exempted Adverse Events** 39](#_Toc520289263)

[8.2 Investigator Responsibilities 39](#_Toc520289264)

[**8.2.1** **Investigator Assessment** 40](#_Toc520289265)

[**8.2.2** **Notification Procedure** 41](#_Toc520289266)

[9 Statistical Considerations 42](#_Toc520289267)

[9.1 Sample Size justification 42](#_Toc520289268)

[9.2 Analysis Plan (Brief) 42](#_Toc520289269)

[10 Data management 43](#_Toc520289270)

[10.1 source data 43](#_Toc520289271)

[10.2 Direct Access to Participant Records 43](#_Toc520289272)

[10.3 Data collection and management responsibilities 43](#_Toc520289273)

[10.4 Data Recording and Record Keeping 43](#_Toc520289274)

[10.5 trial Records retention 44](#_Toc520289275)

[10.6 protocol deviations and violations 44](#_Toc520289276)

[10.7 publication and data sharing policy 44](#_Toc520289277)

[11 Quality Assurance & Control 46](#_Toc520289278)

[11.1 Risk Assessment 46](#_Toc520289279)

[11.2 Central Monitoring at OUCRU CTU 46](#_Toc520289280)

[11.3 On-site Monitoring 46](#_Toc520289281)

[12 Ancillary Studies 47](#_Toc520289282)

[12.1 Pharmacokinetics 47](#_Toc520289283)

[12.2 Health Economics 47](#_Toc520289284)

[13 Regulatory & Ethical considerations 49](#_Toc520289285)

[13.1 Compliance 49](#_Toc520289286)

[**13.1.1** **Regulatory Compliance** 49](#_Toc520289287)

[**13.1.2** **SITE COMPLIANCE** 49](#_Toc520289288)

[13.2 Ethical Conduct of the Study 49](#_Toc520289289)

[**13.2.1** **Ethical Considerations** 49](#_Toc520289290)

[**13.2.2** **Ethical Approvals** 50](#_Toc520289291)

[**13.2.3** **Confidentiality** 50](#_Toc520289292)

[**13.2.4** **Expenses** 51](#_Toc520289293)

[14 Oversight & Trial Committees 52](#_Toc520289294)

[14.1 Trial Management GROUP (TMG) 52](#_Toc520289295)

[14.2 Data Monitoring Committee (DMC) 52](#_Toc520289296)

[15 Finance and insurance 53](#_Toc520289297)

[15.1 Funding 53](#_Toc520289298)

[15.2 INSURANCE 53](#_Toc520289299)

[16 Conflict of Interest 54](#_Toc520289300)

[17 Protocol Amendments 55](#_Toc520289301)

[18 References 56](#_Toc520289302)

[Appendix I - Data Monitoring Committee Membership 58](#_Toc520289303)

[appendix ii - Trial Assessment Schedule 59](#_Toc520289304)

[Appendix III - Toxicity Gradings and Management 62](#_Toc520289305)

Summary of Trial

| Summary Information Type | Summary Details |
| --- | --- |
| Trial Title | A pilot study to assess shortened therapy for hepatitis C infected adults in Vietnam |
| ACRONYM | **SEARCH-1** |
| Trial Design | The study is a single arm, open label trial evaluating strategies for shortening treatment for individuals infected with hepatitis C virus (HCV) based on their early response to directly acting antiviral (DAA) therapy. Participants will be stratified by stage of liver fibrosis to one of two strategies based on virological response at either 48 hours (mild disease) or 14 days (cirrhosis) of treatment. |
| Trial Population/Participants | 103 adults (≥ 18 years) chronically infected with HCV genotypes 1 and 6 |
| Setting | Hospital for Tropical Diseases, Ho Chi Minh City, Vietnam |
| Intervention | Response-guided short course treatment of HCV with sofosbuvir and daclatasvir |
| Study Hypothesis | Shortened HCV treatment courses based on early viral responses to sofosbuvir and daclatasvir therapy will achieve high rates of cure |
| Primary Outcome Measure | Sustained Virological Response (SVR) defined as plasma HCV RNA persistently <LLOQ (lower limit of quantification) through 12 weeks after the end of first treatment without prior failure (see section 2.2) |
| Secondary Outcome Measures | - SVR12 after the end of the combined first and any re-treatment phases (mild patients only) - lack of initial virological response - serious adverse events - grade 3/4 clinical adverse events - adverse events of any grade leading to change in treatment (SOF, DCV or any other medication) - adverse reactions (any grade) (considered definitely/probably/possibly related to SOF or DCV) |
| Participant follow-up | 12 weeks from completion of last therapy |
| Study Duration | 2 years |
| Ancillary Studies | Pharmacokinetics of Sofsobuvir/Daclatasvir  Health economic analysis of HCV treatment |

Trial Schema


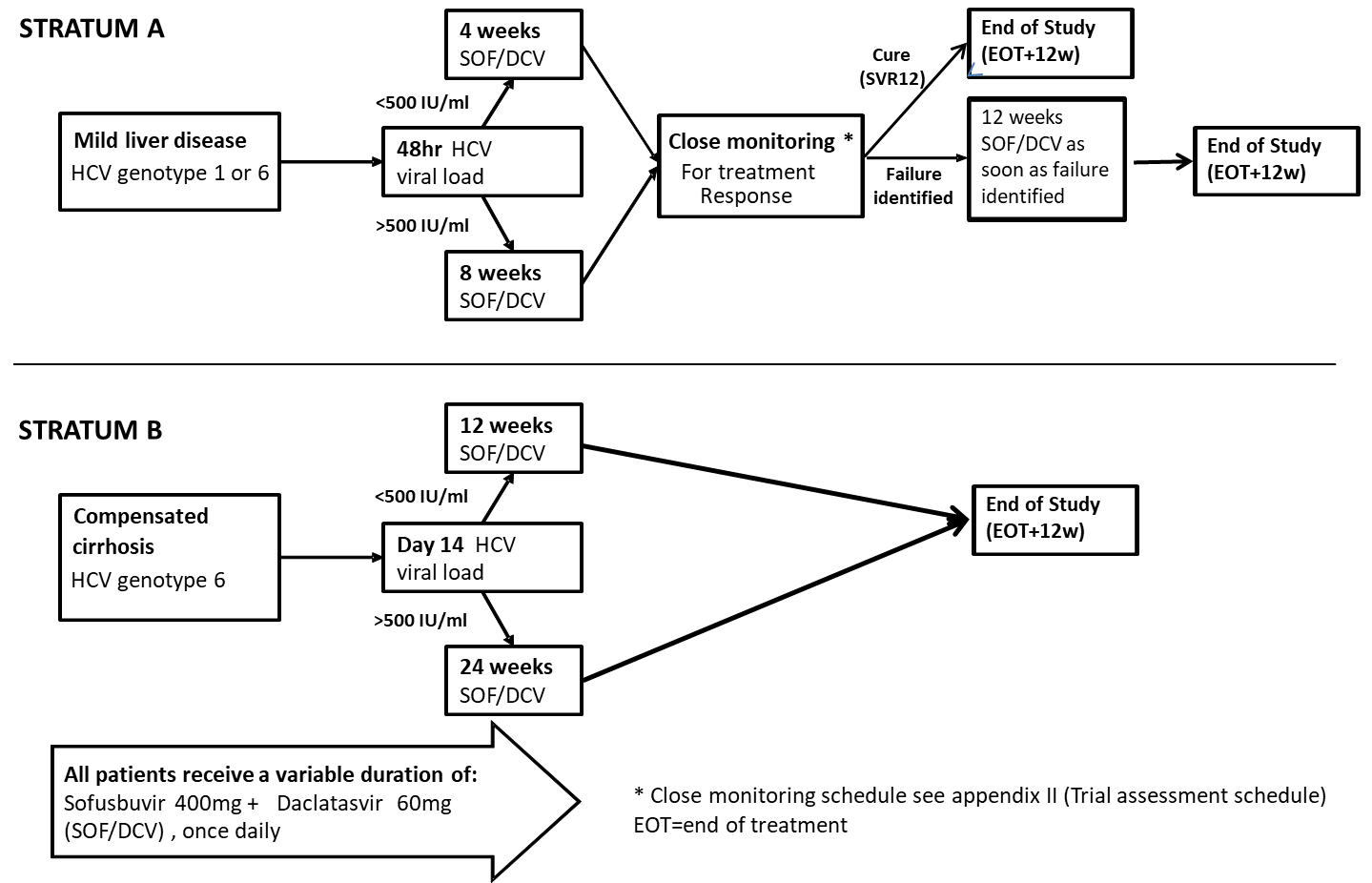


TRIAL ASSESSMENT SCHEDULE

See Appendix II, Trial Assessment Schedule

Abbreviations

| Abbreviation | Expansion |
| --- | --- |
| AE | Adverse event |
| AR | Adverse reaction |
| ART | Antiretroviral therapy |
| ARV | Antiretroviral |
| bid | Bis in die (twice a day) |
| BNF | British National Formulary |
| CF | Consent Form |
| CI | Chief Investigator |
| CI | Confidence interval |
| COM | Clinical Operations Manager |
| CPA, CPB, CPC | Child’s Pugh (classification) A, B, C |
| CPM | Clinical Project Manager |
| CRF | Case Report Form |
| CTU | Clinical Trials Unit |
| DAA | Direct Acting Antiviral |
| DCV | Daclatasvir |
| DCF | Data Clarification Form |
| DAV | Drug Administration of Vietnam |
| DM | Data Manager |
| DMC | Data Monitoring Committee |
| DNA | Deoxyribonucleic acid |
| EASL | [European Association for Study of Liver](https://www.ncbi.nlm.nih.gov/pubmed/?term=European%20Association%20for%20Study%20of%20Liver%5BCorporate%20Author%5D) |
| EC | Ethics Committtee |
| EOT | End of treatment |
| FDA | (US) Food and Drug Administration |
| GCP | Good Clinical Practice |
| HCV | Hepatitis C Virus |
| HIV | Human Immunodeficiency Virus |
| IB | Investigator Brochure |
| ICH | International Conference on Harmonisation of Technical Requirements for Registration of Pharmaceuticals for Human Use |
| IMP | Investigational medicinal product |
| IRB | Institutional Review Board |
| ISRCTN | International Standard Randomised Controlled Trial Number |
| ITT | Intention-to-treat |
| LLOQ | Lower level of quantification |
| LMIC | Lower and middle income countries |
| MedDRA | Medical Dictionary for Regulatory Activities |
| MRC | Medical Research Council |
| MRC CTU at UCL | Medical Research Council Clinical Trials Unit at University College London |
| NIMP | Non-investigational-medicinal product |
| OD | Once daily |
| OxTREC  PD | Oxford Tropical Research Ethics Committee  Pharmacodynamics |
| PI | Principal Investigator |
| PIS | Patient Information Sheet |
| PK | Pharmacokinetics |
| QA | Quality Assurance |
| QC | Quality Control |
| QMAG | Quality Management Advisory Group |
| QoL | Quality of life |
| QP | Qualified Person |
| R&D | Research and Development |
| RCT | Randomised controlled trial |
| REC | Research Ethics Committee |
| RBV | Ribavirin |
| RNA | Ribonucleic acid |
| RVR | Rapid virological response |
| SAE | Serious adverse event |
| SAR | Serious adverse reaction |
| SOF | Sofosbuvir |
| SOP | Standard operating procedure |
| SPC | Summary of Product Characteristics |
| SUSAR | Suspected unexpected serious adverse reaction |
| SVR | Sustained virological response (persistently undetectable) |
| TM | Trial Manager |
| TMF | Trial Master File |
| TMG | Trial Management Group |
| TSC | Trial Steering Committee |
| UAR | Unexpected adverse reaction |
| VL | Viral load |
| WHO | World Health Organization |
| WOCBP | Woman of child-bearing potential |

1. Background information and scientific rationale

**Hepatitis C – the global challenge**

Viral hepatitis is the 7^th^ leading cause of mortality globally, responsible for more deaths each year than tuberculosis, HIV or malaria [[1](#_ENREF_1)]. In contrast to many communicable diseases, its importance to global health has increased significantly over the last 20 years (rising from the 10^th^ leading cause of death in 1990). Just over half of all deaths and disability attributable to viral hepatitis occur in low and low-middle income countries (LMIC).

The greatest driver of this trend is an increasing burden of hepatitis C (HCV). Without an effective HCV vaccine, treatment is a crucial tool in the control of the disease. Until recently, curing HCV required long treatment courses (24-48 weeks) with high rates of side-effects (particularly from injectable interferon), and cure rates were only 40-70% depending on the infecting viral genotype (genotype 1 having previously been considered hardest to treat). The World Health Organisation (WHO) has recently published an ambitious strategy to reduce the burden of deaths from HCV by 60% by 2030, but achieving this will require a substantial increase in treatment access.

**Viral hepatitis in Vietnam**

Vietnam (gross per capita income $1980 in 2015) has an estimated 31,000 deaths from viral hepatitis each year, the 9^th^ highest amongst all countries. In a population of 90 million, an estimated 1.07 million are infected with HCV [[2](#_ENREF_2)]. The HCV epidemic in Vietnam poses particular challenges under-served by current research in the field. The majority of HCV patients are infected with genotype 6 (G6) virus (approx. 54%), a genotype that is relatively uncommon beyond Vietnam’s immediate neighbours (Laos, Thailand and South China) but which accounts for over 5% of the 180 million infected individuals globally [[3](#_ENREF_3)]. HCV G6 has evolved in S.E Asia over >1000 years, in spatially restricted local epidemics, generating enormous viral genetic diversity [[4](#_ENREF_4)]. Despite large Phase III programmes for new HCV treatments, very few G6 patients have been included in these studies (the largest cohort to date is 49 patients) and the evidence base for treatment is small. In addition, the burden of HCV/HIV co-infection is high in Vietnam with an estimated 44,000 HIV/HCV co-infected individuals.


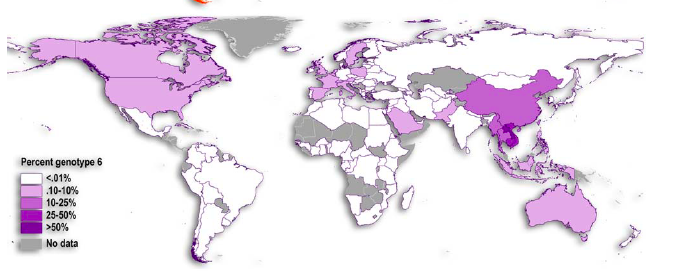


Figure 1 Proportion of HCV infected patients with genotype 6 by country

Treatment of HCV is available in Vietnam, but access even to interferon-based therapy is still relatively limited. The Government currently provides interferon-based therapy and those who have insurance co-pay at least 30% of its cost. Very few patients have access to novel oral treatments, which are only available privately. Although costs will continue to fall with increasing access to generic drugs, treatment will still be prohibitively expensive for most infected individuals. Therefore, any type of treatment will remain beyond the reach of most for the near future.

The work proposed here has its emphasis on infection with HCV genotype 6 (G6), the dominant genotype in Vietnam. This genotype is dominant across Southeast Asia but substantially underrepresented in trials to date

**The ongoing revolution in Hepatitis C treatment**

In the last 3 years, several new drugs for HCV have been approved and entered clinical practice, with further drugs arriving in the next 2 years [[5](#_ENREF_5)]. These new directly acting antivirals (DAAs) are replacing interferon-based regimens and have very much improved side-effect profiles. They offer cure rates over 90% with significantly shorter durations of therapy (typically 12 weeks). More recently developed treatment combinations also have broad genotypic activity (G1-6). Pangenotypic agents would reduce the need for complex diagnostics and simplify models of care, which must currently target specific drugs to specific genotypes.

These advances have the potential to overcome many of the major barriers to treatment in LMICs. However, two remain. Firstly, and most importantly, is cost. One leading treatment regimen (sofosbuvir/ledipasvir) has a US list price of ~USD84,000, placing it beyond the reach of many people even in high-income countries. Through voluntary licensing agreements with generic manufacturers the same treatment will be available in LMIC for approximately USD1000, potentially falling further once competition develops between generic manufacturers. However, even these costs will remain too high for many individuals. Governments in LMICs will struggle to afford widespread access to treatment given the numbers infected. Secondly, even if they can afford it, 12 weeks of therapy will be challenging for many patients with HCV, who often struggle to engage with care. However, it is curing such hard to reach populations (including injecting drug users, prisoners and the homeless) that will have the greatest impact on the HCV epidemic in terms of curtailing transmission.

**Stratified medicine and Hepatitis C**

Stratified (or precision) medicine, tailoring individual treatment based on clinical or laboratory findings, has long been part of the management of HCV-infected individuals in the era of interferon-based therapy. Until 2011, treatment consisted of a combination of injectable interferon and oral ribavirin, the recommended duration of therapy varying between 24-48 weeks depending on the viral genotype present. Host polymorphism in the IL28B gene was associated with differing cure rates in genotype 1 infection and was used to guide treatment in some clinics. On-treatment response (rapid virological response, RVR) 4 weeks after starting treatment was also used to tailor individual therapy. Viral genotype has remained an important part of treatment decision making with 1^st^ (e.g. boceprevir, telaprevir) and 2^nd^ generation (e.g. sofosbuvir, daclatasvir, ledipasvir) DAAs, and may be needed even with the 3^rd^ generation of more pangenotypic treatments (e.g. ravidasvir, velpatasvir).

However, given vastly different on-treatment viral dynamics and much higher cure rates, a new evidence base is needed to inform stratified approaches in interferon-free therapy. This is particularly important in many health systems where the cost of treatment (paid per tablet) is a limiting factor to access to therapy. The UK STOPHCV-1 trial (ISRCTN 37915093), supported by NIHR and MRC, and led by the MRC-funded STOPHCV stratified medicine consortium (including four of the co-investigators), is one of the first post-licensing trials aiming to define the optimum treatment for individual patients. This trial includes only genotype 1 infection and relies on next-generation sequencing methods developed by the consortium for HCV which are providing new insights into treatment response. Further trials are needed in patients infected with all viral genotypes (specifically in Genotype 1 and 6 which are common in Asia), evaluating strategies that may be appropriate for LMIC.

In addition to host IL28B genotype and viral genotype, this study will enable us to investigate additional stratification parameters that may play an important role in treatment outcome. The MRC STOP-HCV study team have recently developed new methods for generating and integrating full genome HCV sequencing with host genetic polymorphisms (Azim *et al* Nature genetics). This works has shown that the host genome “shapes” the viral genome and that distinct host and viral polymorphisms are associated with important clinical parameters such as HCV viral load. We will apply these sequencing technologies in this study to assess the role of viral and host genetics in the treatment outcome of viral genotypes that are common in Asia. In theory, host and viral genetics and other serum biomarkers (which may be measured in a targeted approach e.g. IL-10 and vitamin D, or through metabolomic/proteomic screening), may be particularly suited for predicting successful treatment response when HCV therapies are shortened. For these reasons blood will be evaluated for host and viral genetic parameters and stored for the future evaluation of serum biomarkers.

**Increasing options for treatment of chronic hepatitis C**

Several new interferon-free treatment combinations for HCV have come to market in the US and Europe representing a maturation and stabilization of the HCV treatment field. Four combinations have been approved by the Food and Drug Administration (FDA) in 2016/7 - sofosbuvir/velpatasvir (a combination of an NS5B and an NS5a inhibitor), sofosbuvir/velpatasvir/voxilprevir (addition of a protease inhibitor), pibrentasvir/glecaprevir (NS5a/protease inhibitor) and MK-3682/elbasvir/grazoprevir (NS5a/NS5b/protease inhibitor). Whilst all these combinations are promising options and should have good activity against G6 (although data is limited), access to these treatments is likely to be limited in the short-medium term, particularly in LMIC.

However, most recent EASL guidelines (2016) also recommend sofosbuvir/daclatasvir for genotype 6 (12 weeks for cirrhotic and non-cirrhotic) (in addition to sofosbuvir/velpatasvir and sofosbuvir/ledipasvir). American guidelines (December 2017) recommend glecaprevir/pibrentasvir first-line for patients with (12 weeks) or without (8 weeks) cirrhosis. Sofosbuvir/velpatasvir and sofosbuvir/ledipasvir are also recommended combination treatments for genotype-6. However, another combination of drugs from the same two classes, sofosbuvir/daclatasvir, is not widely reimbursed in the US due to commercial considerations (made by two different companies) and is therefore not considered within their guidelines. Most recent Asian guidelines (2015) recommend 12 weeks of sofosbuvir/daclatasvir as an option for genotype-6 non-cirrhotic patients and 24 weeks sofosbuvir/daclatasvir as an option for cirrhotic patients. **However, in practice data on genotype-6 patients with all these combinations is limited (see below for summary of data of sofosbuvir and daclatasvir).**

The combination of sofosbuvir (SOF) and daclatasvir (DCV, another NS5A inhibitor) was the first combination with broad genotypic activity to be evaluated in late stage clinical trials. The combination showed good efficacy and tolerability. However, the two agents were developed by different originator companies and one of those, Gilead, elected to pursue development of its own NS5A inhibitor (velpatasvir) in preference to supporting daclatasvir. As a result, sofosbuvir/daclatasvir is not a commercially viable option in most high-income countries. However, both drugs are listed on the WHO’s Essential Medicine List and there is active competition amongst generic manufacturers to supply treatment to LMICs. Prices for sofosbuvir/daclatasvir are falling and, at the time this protocol was written, were already below $300 for a 12 weeks course from some manufacturers. Imminent affordable generic production will mean SOF/DCV will be a key economically viable treatment option for LMIC. However, currently the evidence base for treatment is limited, particularly for genotype 6 infections.

**Evidence of efficacy and safety of sofosbuvir/daclatasvir for treatment of chronic HCV infection**

Recommendations for treatment with sofosbuvir and daclatasvir are based on registration trials of SOF/DCV (particularly the ALLY series of trials) and the Phase II trial of SOF/DCV (AI444-04 [[6](#_ENREF_6)]). SOF/DCV showed high efficacy with a good safety profile across a range of clinical populations relevant to this study.

**Recommended treatment in non-cirrhotic populations**

The AI444-04 trial studied 211 patients with genotypes 1-3 without cirrhosis. Treatment naïve genotype 1a and 1b patients had 5 different therapies (i) SOF for 7 days, then DCV + SOF for 23 weeks (n = 15) (ii) DCV + SOF for 24 weeks (n = 14) (iii) DCV + SOF + ribavirin (RBV) for 24 weeks (n = 15) (iv) DCV + SOF for 12 weeks (n = 41) (v) DCV + SOF + RBV for 12 weeks (n = 41). Of the 167 patients infected with GT-1, 164 (98%) achieved SVR12; this total included 84 of the 85 patients given 24 weeks of treatment and 80 of the 82 patients given 12 weeks of treatment. The proportions of GT-1a- and GT-1b-infected patients who achieved SVR12 were 98% (129 of 132 patients) and 100% (35 of 35 patients), respectively.

The recommended treatment for patients with genotype 1 infection and no cirrhosis is 12 weeks of sofosbuvir and daclatasvir (without ribavirin).

**Genotype 6 and SOF/DCV**

The programme of ALLY trials only included one patient with genotype 6 infection; this patient was in the post-transplant cohort of the ALLY-1 trials [[7](#_ENREF_7)]. This patient achieved SVR12 with 12 weeks of SOF/DCV and ribavirin. The French Compassionate Use Programme enrolled 5 genotype 6 patients with advanced liver fibrosis. Patients were treated with 24 weeks of SOF/DCV with ribavirin added at the physician’s discretion. 3/5 patients had cirrhosis and 5/5 achieved SVR12 (Bristol Myers Squibb, personal communication). As such whilst SOF/DCV is recommended as an option for treatment for genotype 6 in clinical guidelines [[8](#_ENREF_8)], there is no specification made for duration of therapy.

Real-world data is emerging: recent data of SOF/DCV in genotype-6 shows high cure rates [[9](#_ENREF_9)]. 120/127 (94%) genotype-6 patients achieved SVR12 with 12 weeks of treatment. Cure rates were 92% (52/59) with 12 weeks’ treatment even in the hardest to treatment population with cirrhosis.

**Recommended treatment in cirrhotic populations**

The ALLY-1 trial enrolled 60 patients with advanced cirrhosis. 12 (20%) patients were classified as Childs Pugh A (CPA), 32 (53% as Childs Pugh B (CPB), and 16 (27%) as Childs Pugh C (CPC). 45 (75%) were genotype 1a/1b, the remainder representing genotypes 2-5 (no genotype 6). It is this absence of genotype 6 data that will be addressed in stratum B. All received 12 weeks of sofosbuvir and daclatasvir with dose-adjusted ribavirin for 12 weeks. Overall 50/60 (83%) achieved SVR12. SVR12 was achieved in 11/12 (92%) of those with CPA cirrhosis.

Based on these findings and those of AI-444-04 (above), current recommended treatment for patients with compensated cirrhosis (Childs Pugh A or B) is either 12 weeks SOF/DCV with ribavirin or 24 weeks without ribavirin.

**SOF/DCV treatment for less than 12 weeks**

There are very limited data on the efficacy of SOF/DCV at durations less than 12 weeks. The largest study to date is the ALLY-2 trial [[10](#_ENREF_10)] limited to the treatment of HIV/HCV co-infected individuals and for this reason HIV co-infected patients are not included in stratum A. 76% (31 of 41) of individuals with genotype 1 infection receiving a shortened duration of therapy (8 weeks; not selected based on treatment response or baseline characteristics) achieved SVR12. The question addressed within this protocol is whether early on treatment responses can be used to increase cure rates with shortened therapy.

There is one study to date of response guided therapy published with sofosbuvir/daclatasvir [[11](#_ENREF_11)]. Treatment was shortened from 12 to 8 weeks in an Egyptian genotype 4 population when viral load was undetectable at day 14. The shortening strategy was found to be non-inferior to the standard of care. There are no studies seekinn to identify patient groups who may be cured with less than 8 weeks of therapy with sofosbuvir/daclatasvir. However, one study from Lau et al [[12](#_ENREF_12)] in a highly selected population genotype 1b, female, with low BMI, found 100% cure rate (18/18) with 3 weeks of triple-drug combination treatment (not SOF/DCV) for those patients with a rapid virological response (<500 IU/ml at 48h). The hypothesis tested in this trial is that those patients responding in a similar way to SOF/DCV will be cured with shortened therapy. Whilst the Lau et al paper suggests high cure rates may be achieved with 3 weeks of therapy, a four week duration will be used in this study to provide greater chance of successful treatment.

There is very little data available on retreatment of patients receiving shortened or standard 12 week courses of NS5a-inhibitor-containing treatments such as daclatasvir, velpatasvir or ledipasvir. Options including glecaprevir/pibrentasvir and sofosbuvir/velpatasivir/voxilaprevir are not yet available in Vietnam. In the small studies looking at re-treatment with NS5a/NS5b combinations, emergent resistance associated variants (RAVs) were less likely to occur in those who received shorter courses of first-line treatment [[13](#_ENREF_13)] (SYNERGY)[[14](#_ENREF_14), [15](#_ENREF_15)]. Patients in the SYNERGY study [[16](#_ENREF_16)] who failed first-line treatment still achieved 90% SVR12 when retreated with sofosbuvir/ledipasvir without ribavirin for 12 weeks [[15](#_ENREF_15), [17](#_ENREF_17)].

**Risks and benefits at patient and societal level**

The primary benefit of participation for individual patients in this trial is that >90% of individuals will achieve cure of hepatitis C without interferon-based treatment within the protocol. For many, interferon will have been a barrier to accessing and/or adhering to treatment previously. Barriers to accessing treatment are likely to remain even with increasing availability of directly acting antivirals (DAAs), as demand for treatment is expected to exceed the capacity to deliver it in setting such as Vietnam. The primary outcome measure (sustained virological response 12 weeks after end of treatment, SVR12) has been associated with significant reductions in all-cause mortality, cirrhosis and liver cancer [[18](#_ENREF_18)], and so achieving cure will significantly reduce individual patient’s future risks of these events. The study will use licensed medications that have been recommended as part of the WHO Essential Medicines list since 2015.

1. trial design and endpoints
   1. Description of trial design

The study is a single arm, open label trial with the objective of identifying successful strategies for shortening treatment for individuals chronically infected with hepatitis C based on their early response to therapy.

It will test the hypothesis that early responses to treatment with sofosbuvir and daclatasvir will identify a group of individuals able to achieve high cure rates with shortened courses of treatment. Participants will be stratified by stage of liver disease to one of two strategies based on virological response after either 48 hours (non-cirrhotic) or 14 days (cirrhosis) of starting treatment.

- 1. trial endpoints

Primary endpoint

The primary endpoint for the trial is Sustained Virological Response (SVR12) defined as plasma HCV RNA <LLOQ (lower limit of quantification, <15 IU/ml) 12 weeks after the end of first treatment without prior failure defined as either:

i. two consecutive measurements of HCV RNA >LLOQ (taken at least one week apart) after two consecutive visits with HCV RNA <LLOQ, at any time, with the latter confirmatory measurement also being >2000 IU/ml

ii. two consecutive measurements of HCV RNA (taken at least one week apart) that are >1 log10 increase above HCV RNA nadir on treatment and >2000 IU/ml, at any time.

Cure is defined using the LLOQ, <15 IU/ml, 12 weeks after end of treatment (EOT). However, failure before this timepoint is defined using a slightly higher threshold because patients have been observed to achieve cure despite having low-level viraemia at the EOT or shortly after, and so they do not need re-treatment to achieve cure. In practice any patient with low-level viraemia <2000 IU/ml either cures or viral load rises above this level. This will be carefully reviewed by the Data Monitoring Committee. This is the same definition as used in the UK STOP-HCV-1 trial ISRCTN 37915093), where the Data Monitoring Committee has similarly reviewed individual patient viral load trajectories and has not raised any concerns.

Secondary endpoints

Secondary endpoints for the trial are

- SVR12 after the end of the combined first and any re-treatment phases (mild patients only)
- lack of initial virological response (<1 log_10_ decrease in HCV viral load from baseline)
- serious adverse events
- grade 3/4 clinical adverse events
- adverse events of any grade leading to change in treatment (SOF, DCV or any other medication)
- adverse reactions (any grade) (considered definitely/probably/possibly related to SOF or DCV)

1. Participant identification

There will be no exceptions to eligibility requirements at the time of recruitment. Questions about eligibility criteria should be addressed prior to attempting to enrol the patient – clarifications may be sought from the trial team.

The eligibility criteria are the standards used to ensure that only medically appropriate participants are considered for this study. Participants not meeting the criteria should not join the study. For the safety of the participants, as well as to ensure that the results of this study can be useful for making treatment decisions regarding other participants with similar diseases, it is important that no exceptions be made to these criteria for admission to the study.

Participants will be considered eligible for enrolment in this trial if they fulfil all the inclusion criteria and none of the exclusion criteria as defined below.

Recruitment will take place from clinics within the Hospital for Tropical Diseases in Ho Chi Minh City.

- 1. Inclusion Criteria

**For all participants enrolled in Stratum A (mild disease) or Stratum B (cirrhosis) inclusion criteria are**

1. Aged ≥ 18 years
2. At least one detectable viraemia prior to the screening visit (by quantitative HCV RNA, qualitative assay or HCV genotype), with no intervening undetectable results
3. Plasma HCV RNA >LLOQ at screening
4. BMI >=18kg/m^2^
5. Laboratory tests: creatinine clearance (estimated using Cockcroft-Gault) >=60ml/min, international normalised ratio (INR) <1.5xULN
6. Written informed consent obtained from the patient.

**For Stratum A, additional inclusion criteria are**

1. Infected with HCV genotype 1 (all subtypes) or genotype 6 (all subtypes)
2. Mild liver disease: No evidence of significant liver fibrosis resulting from any aetiology, defined as

EITHER
(a) Fibroscan* score ≤7.1kPa, equivalent to F0-F1 [[19](#_ENREF_19)], within 180 days prior to planned enrolment,

OR
(b) biopsy consistent with mild fibrosis (Ishak score <=2/6) within 180 days prior to planned enrolment

**For Stratum B, additional inclusion criteria are**

1. Infected with HCV genotype 6 (all subtypes)
2. Compensated Cirrhosis: Evidence of compensated cirrhosis resulting from any aetiology defined as

EITHER
(a) Fibroscan* score ≥10.1kPa within 180 days prior to enrolment

OR
(b) biopsy consistent with cirrhosis (Ishak score >=5/6 or equivalent) within 180 days prior to enrolment)

OR
(c) Imaging (Ultrasound or CT or MRI) reported as showing cirrhosis

1. Childs-Pugh Score ≤7 according to following scoring system

* Fibroscan must be a valid result (based on at least 10 readings) performed by an experienced (as evidenced by CV and/or training logs) technician.

- 1. Exclusion Criteria

1. Previous Hepatitis C (treatment with DAAs, and/or pegylated-interferon and/or ribavirin)
2. HIV infected
3. Solid Organ Malignancy within 5 years prior to screening
4. Any condition in the judgement of the investigator which might limit the patient’s life expectancy within the duration of the study
5. Any disorder or circumstance which in the opinion of the investigator may have a significant negative impact on the ability of the patient to adhere to the trial regimen
6. HBsAg positive
7. Disorder which may cause ongoing liver disease including, but not limited to, ongoing alcohol misuse
8. Currently receiving medication known to interact with study medications for which dose adjustment for daclatasvir or sofosbuvir would be recommended in the Summary of Product Characteristics
9. Use of other investigational products in clinical studies within 60 days of screening
10. History of severe pre-existing cardiac disease, including unstable or uncontrolled cardiac disease, in the previous six months
11. Patients currently using amiodarone or other anti-arrhythmics (including patients with permanent pacemakers).
12. Pregnancy
13. Symptomatic Haemoglobinopathy (e.g., thalassemia major, sickle-cell anaemia).
14. Unlikely to be able to attend follow-up visits for any reason, as judged by the attending physician
    1. Number of Participants

In total, 103 participants will be recruited, stratified by stage of liver disease. 60 participants will be recrutied with mild liver disease (30 genotype 1, 30 genotype 6). 43 participants will be recruited with compensated cirrhosis (genotype 6 only)

1. REGISTRATION AND RECRUITMENT
   1. Recruitment and retention strategies

Participants will be recruited in Ho Chi Minh City, Vietnam at the Hospital for Tropical Diseases. Potential participants will be identified and approached by clinic staff to participate in the trial. In addition, appropriate patients who are part of the Hepatitis Cohort will be approached for participation in line with previous permissions. Recruitment activities will only occur in an out-patient hospital setting and no activities will be carried out outside of the participating hospital. The target sample size of 103 participants will be enrolled.

To ensure an optimal retention rate, participants will be contacted by phone to remind them of their next visit. In addition, participants who have missed a visit will be contacted by phone for a maximum of three times after which a maximum of three home visits can be conducted.

- 1. Screening Procedures & Pre-enrolment Investigations

All potential participants will be identified for screening in the out-patient department of the HTD, HCMC. As part of their standard care participants will have routine laboratory tests, including liver function tests; hepatitis C viral load and genotyping; HIV tests, and Fibroscan. These tests will be used to identify participants who may be suitable for screening.

The name and date of birth of every adult screened for the trial will be added to the site Screening & Enrolment Register, together with allocated trial number if subsequently enrolled, or the reason the participant was not enrolled. The Screening & Enrolment Register will be kept in a secure location and will be the responsibility of the site Principal Investigator.

A fibroscan or biopsy result will be valid if conducted within the 180 days preceding enrolment. The screening visit may be conducted up to 60 days prior to recruitment, providing that results of blood tests to confirm eligibility in terms of laboratory abnormalities and HCV VL are available.

- 1. informed consent

The site PI or an appropriately trained doctor must seek written informed consent from patients to enter into the trial in their own language. Individuals trained and responsible for taking consent will be documented on the trial’s Delegation Log (with signatures). Consent will be sought after explanation of the aims, methods, benefits and potential hazards of the trial and BEFORE any trial-specific procedures are performed or any blood is taken for the trial, including for the screening assessment.

The doctor taking consent must make it completely and unambiguously clear that the participant is free to refuse to participate in or withdraw from all or any aspects of the trial, at any time and for any reason, without incurring any penalty or affecting their subsequent treatment. This will be stated explicitly in the participant information sheet.

Signed consent forms must be kept by the investigator and documented in the case record form (CRF) and a copy given to the participant.

- 1. Co-enrolment Guidelines

Treatment with any other investigational product within 60 days prior to screening is an exclusion criteria, and therefore co-enrolment with a previous investigational medicinal product (IMP) trial will generally not be allowed, unless the patient is in a follow-up phase. Before enrolment into the study of a patient already participating in a previous trial (not of an investigational product), the site investigator must confirm with the investigators of the previous trial that co-enrolment is allowed. Co-enrolment in the study of patients already participating in an observational study is allowed.

1. TReatment of PARTICIPANTS

Each participant will be treated with the same drug combination (sofosbuvir 400mg and daclatasvir 60mg daily), but with differing durations of therapy. The study design is open-label; bias will be minimised by using objective laboratory measurements to determine duration of therapy and to assess treatment outcomes .

Any patient in the mild disease cohort who experiences failure of first-line treatment will initiate re-treatment as soon as possible after failure is identified. Failure of first-line treatment during the study is defined as

1. two consecutive measurements of HCV RNA > LLOQ (taken at least one week apart) after two consecutive visits with HCV RNA <LLOQ, at any time during the study follow-up period, with the latter confirmatory measurement also being >2000 IU/ml OR
2. two consecutive measurements of HCV RNA (taken at least one week apart) that are >1 log10 above the nadir on treatment and >2000 IU/ml, at any time during the study follow-up period.

Failure as defined forms part of the definition of the primary endpoint, see section 2.2.

Patients will have ultrasound and AFP monitoring in line with Vietnamese national guidelines; that is at study entry, end of treatment and 12 weeks after the end of treatment. Study follow-up completes at 12 weeks after the end of treatment, or re-treatment, for all patients. Following study participation, all patients will return to normal standard of care follow-up within the hospital where they will undergo follow-up for up to 2 more years according to national guidelines.

- 1. Treatment Schedule

The duration of first-line SOF/DCV treatment will be determined by the initial response to treatment, as outlined in trial schema above

**STRATUM A (mild liver disease, G1 and 6 HCV, Ultrarapid Repsonse Guided Therapy)**

All participants will receive a minimum of 4 weeks of therapy. All indiviudals will have HCV viral load assayed at enrolment and 24 and 48 hours after initiation of therapy (first dose to be taken in the clinic at D0). For participants with viral load <500IU/ml (as measured by Cobas assay, Roche) at 48 hours (Day 2 visit), treatment will be completed at 4 weeks. All other participants will receive 8 weeks of therapy. Following cessation of first line therapy (at the end of treatment (EOT) visit), all participants will have HCV viral load assayed twice weekly for four weeks so that the kinetics of any viral rebound post-EOT can be captured in detail. Participants meeting the definitions of first line failure above will be eligible for retreatment according to protocol at any time after EOT +2 weeks.

**STRATUM B (compensated cirrhosis, G6 HCV only, Response guided therapy)**

All participants will receive a minimum of 12 weeks of treatment. Those participants who have achieved virological suppression (VL <500IU/ml, as measured by Cobas assay, Roche) at the time of their D14 visit or sooner after treatment initiation will complete therapy at 12 weeks. Those who do not achieve virological suppression will continue to complete 24 weeks of therapy.

- 1. Protocol Treatment Discontinuation

In consenting to the trial, participants are consenting to trial treatment, trial follow-up and data collection.

Treatment (or retreatment) will be discontinued if any of the following occurs:

- toxicity which the investigator considers impedes the ability of the patient to continue intervention and/or the product information for the drug(s) contains a directive on discontinuation in the event of this specific toxicity
- participant requests for any reason to withdraw consent for treatment
- virological failure as defined above (move to retreatment if first-line failure and mild disease)

However, any such patient discontinuing study treatment (or retreatment) will be invited to continue follow-up “off study drug, on study” in order to obtain data on overall cure and adverse events. Participants should only discontinue study follow-up if they explicitly withdraw consent to continue to attend study visits. In this situation, participants may still contribute data collected at their routine clinic visits to the trial, providing they are willing to do so (“off study, follow-up through medical records only”).

1. Assessments & Follow-up
   1. Trial Assessment Schedule

See Appendix II, Trial Assessment Schedule.

All participants will be followed by the site clinic teams for 12 weeks after the end of first-line treatment or re-treatment (where relevant) for evaluation of virological response and toxicity. Outcome measures will be assessed at clinic visits (see Trial Assessment Schedule). For the mild disease cohort, the first DAA dose should be taken in the clinic and the blood draws on day 1 and day 2 should be taken within ±3 hours of 24 and 48 hours post this first dose wherever possible. Other on treatment visits should occur within ±1 days wherever possible, and the post end-of-treatment (EOT) visits within ±3 days. However, if this is not possible, a visit (with all the appropriate tests and evaluations) should still take place outside this window to ensure that trial outcomes are ascertained as accurately as possible in real-time.

All those recording clinical data will be identified and receive appropriate training and sign the Delegation Log. Clinical data will be obtained through consultation with the participant, their medical team, or their medical records. Laboratory measures and resource utilisation will be extracted from participant notes/electronic records; study nurses will administer quality of life questionnaires to participants.

Any additional visits or diagnostic/laboratory tests needed for patient management should occur as required at the discretion of the treating physician. Results from these investigations should be recorded on CRFs, but only the investigations specified in the Trial Assessment schedule are required in all participants.

- 1. Procedures for Assessing Efficacy

The primary means of assessing efficacy is the HCV viral load. HCV viral load will be assayed locally in line with existing clincial practice. This will also enable swift detection of virological failure and move to re-treatment for participants in the mild disease cohort.

HCV viral resistance will be measured within the SEARCH consortium retrospectively in batches during the trial. This will be done using next-generation sequencing approaches. Viral genotype will also be characterised using these assays enabling an assessment of whether treatment failure is a genuine recurrence (same genotype, closely genetically related) or a new infection (different genotype, or same genotype but genetically too distant to be compatible with within-host evolution). Summary resistance data will be shared with the local research team.

In terms of incidental findings from these next generation sequencing studies, we will follow the principles that health related findings (HRFs) identified during viral genetics studies that are associated with serious and treatable infectious disease WILL be fed back to participants. The only pathogens falling within this category of HRFs at the current time are HIV and Hepatitis. HRFs identified during host genetics studies will NOT be fed back to participants.

- 1. Procedures for Assessing Safety

Laboratory assessments to ensure patient safety will be performed routinely, and additional safety blood tests or investigations may be performed to investigate symptoms or monitor emergent laboratory test abnormalities as clinically indicated. Laboratory abnormalities that require medical or surgical intervention or lead to an interruption, modification, or discontinuation in study medication will be recorded as an AE, as well as an SAE (if applicable).

Clinical AEs and SAEs will be elicited (together with relationship to study medications) throughout the study at the regular clinical assessments, through consultation with the participant, their medical team, or their medical records.

In addition, laboratory or other abnormal assessments (eg, electrocardiogram, x-rays, vital signs) that are associated with signs and/or symptoms must be recorded as an AE or SAE if they meet the definition of an AE or SAE (see Section 8). If the laboratory abnormality is part of a syndrome, the diagnosis or syndrome will be recorded (eg, anaemia), in preference to the laboratory result. The severity grading of AEs will be assessed as Grade 1, 2, 3, or 4 using the Common Toxicity Criteria grading scale (see Appendix III).

All AEs should be recorded in the patient’s source documents, only those which are:

- serious,
- reactions (any grade) considered definitely/probably/possibly related to SOF or DCV,
- clinical grade 3 or 4,
- any grade leading to change in treatment (SOF, DCV or any other medication)

need to be reported on trial CRFs.

See Safety Reporting Section 8 and Section 8.2 in particular for investigator’s responsibilities regarding assessment of seriousness, causality and expectedness, and reporting to OUCRU CTU.

Any participant experiencing an AE or SAE will be followed up until resolution or stabilisation of the AE. For AE/SAEs leading to discontinuation of study medication, patients will continue to be followed up “off study drug, on study”. Any participant who wishes to withdraw from study visits after an AE/SAE leading to discontinuation of study medication must still be followed up in routine clinics for 12 weeks following cessation of therapy.

- 1. Other assessments

**Health economics and cost-effectiveness**

Using established models describing the progressive stages of chronic HCV, we will aim to translate the outcomes of the trial into an estimated number of disability-adjusted life years (DALYs) averted. This will follow the approach used within the 2010 global burden of disease study. In addition, we will aim to administer the EQ5D-3L quality of life questionnaire to all of the enrolled patients at baseline (before treatment), midway during treatment, and after treatment (at the EOT+12 week visit). This data will allow us to measure the number of quality-adjusted life-years (QALY) saved due to the therapy.

**Pharmacokinetics**

Venous plasma samples will be collected for sofosbuvir, and its main metabolite GS-331007, as well as daclatasvir for pharmacokinetic evaluations. Dense pharmacokinetic samples will be collected in 40 patients with mild liver disease. Five samples will be collected in each patient after the first dose and at day 28, resulting in a total of 10 samples per patient. Sparse pharmacokinetic samples will be collected in all patients. A total of three samples per patient will be collected randomly at D7, D14 and the last day of treatment (either D28 or D56). The pharmacokinetic properties of these drugs will be evaluated with respect to viral dynamics in order to characterise the pharmacokinetic-pharmacodynamic properties of sofosbuvir and daclatasvir.

**Blood sampling and laboratory analysis**

Blood samples to monitor safety and response to therapy will be drawn from each subject according to trial assessment schedule. Samples for hematological and biochemical tests will be sent to HTD clinical laboratory (ISO 15189;2012). Any critical values identified in hematology or biochemistry paprameters will immediately be reported to the study physician. HCV viral load will be determined by Cobas TAqMan HCV test v2.0 (Roche Molecular diasgnostic) (LOD 15IU/ml) throughout the study. HCV genotype will be determined by NS5B, Core, 5’ UTR sequencing.

Additional blood samples for research purposes, as detailed in the trial assessment schedule, will be collected, stored, and anonymised. These samples will include plasma, serum, DNA, RNA and whole blood. Samples will be collected according to standard local procedures. Samples for virological, genetics and immunological assays will be processed and stored at -80C or in liquid nitrogen at OUCRU laboratory. Chain of custody of all samples will be maintained during the study period and therafter as approved by the EC. IL28B and other SNPs will be determined by chip tecnology available at Oxford University. Full length viral sequencing will be done to study variation in the viral genome associated with treatment come. Host genetic analysis including SNP in IL28B gene and other known SNPs will be studied and their association with treatment outcome will be analyzed. Samples will be analyzed for biomarkers for HCV infections, liver disease and their association with clinical outcome, in particular the cytokines IL-10, IL-6 and TNF[[20](#_ENREF_20)] and IP-10 [[21](#_ENREF_21)]. All testing will be done by trained technicians following GCLP standard. All samples will be stored at OUCRU and will be shared with investigators in Oxford University for analysis as necessary. Blood samples may be shipped for experimental work to laboratories outside Vietnam (to Oxford University and Imperial College who are investigators in this study).

All data arising from this collaborative project will remain the shared property of HTD, collaborators, and OUCRU. Use of these samples and data beyond the stated objectives of this study will require the joint agreement of all the Investigators. Additional investigations will only be performed with the approval of the appropriate ethical committees and the endorsement of the responsible investigators.

- 1. Early Stopping of Follow-up

If a participant chooses to discontinue their trial treatment, they should always be invited and encouraged to continue follow-up “off study drug, on study”. If they do not wish to remain on trial follow-up, however, their decision must be respected and the patient will be withdrawn from the trial. However, in this situation, the participant should be asked whether or not they are willing to provide follow-up through their routine records (that is, not to attend study-specific visits but to allow data collected within the clinic to be used for trial comparisons). The OUCRU CTU should be informed of the participant’s decision regarding withdrawal (no further data of any kind, or follow-up through routine records) in writing using the appropriate CRF. Prior to withdrawing from the trial, the participant will be asked to have assessments performed as appropriate for the final EOT+12 week visit although they would be at liberty to refuse any or all individual components of the assessment.

If a participant withdraws from the trial, the medical data collected during their previous consented participation in the trial will be kept and used in analysis. Consent for future use of stored samples already collected can be withdrawn when leaving the trial early (but this should be discouraged and should follow a discussion). If consent for future use of stored samples already collected is withdrawn, then all such samples will be destroyed following the policies of the institution where the samples reside at the time.

Participants may change their minds about stopping trial follow-up at any time and re-consent to participation in the trial, including taking repeat samples.

Participants who stop trial follow-up early will not be replaced, as the total sample size includes adjustment for losses to follow-up.

Consent will be sought for long-term follow-up through routine records in order to ascertain long-term impact of treatment on morbidity and mortality including cirrhosis and hepatocellular carcinoma.

- 1. Loss to Follow-up

In the statistical analysis, a participant will be classified as ‘lost to follow-up’ if they have not been seen at the final EOT+12 week visit within a [-6,+6] week window.

6.7 Completion of protocol follow up

The trial will end at the final first-line or re-treatment EOT+12 week visit of all enrolled participants. Because the trial may contain a re-treatment phase, this may not necessarily be the final EOT+12 week visit of the last enrolled participant.

Following study participation, all patients will return to standard of care follow-up within the HTD clinic. This routine follow-up is not part of study procedures.

1. investigational medicinal product
   1. Introduction

All trial participants will use the same interventional medicinal products, sofosbuvir 400mg and daclatasvir 60mg. Both are licensed agents and have been used previously in a range of registration trials (see section above). The duration of therapy will differ for individual participants. Both drugs must be used together.

Both sofosbuvir and dalclatasvir of the trial will be sourced from Pharco Ltd, Egypt. Pharco are a generic manufacturer who have provided generic drugs worldwide, including to a recent real-world study treating 18,572 patients in Egypt with sofosbuvir/ daclatasvir (predominantly genotype-4, the most common genotype in Egypt) achieved >95% cure [[22](#_ENREF_22)].(Investigators Brochure provide separately)

- 1. SOFOSBUVIR (SOF)
     1. **PRODUCT**

The product is SOF 400mg taken once a day. Participants should be instructed to swallow the tablet whole **with food**. Participants should be instructed that if vomiting occurs within 2 hours of dosing an additional tablet should be taken. If vomiting occurs more than 2 hours after dosing, no further dose is needed.

**Side-effects of SOF and DCV**

Adverse reactions are listed in by regimen, system organ class and frequency: very common (≥1/10), common (≥1/100 to <1/10), uncommon (≥1/1,000 to <1/100), rare (≥1/10,000 to <1/1,000) and very rare (<1/10,000).

| System order class | SOF+DCV |
| --- | --- |
| **Psychiatric disorders** |  |
| Common | Insomnia |
| **Nervous system disorders** |  |
| Very common | Headache |
| Common | Dizziness, migraine |
| **Gastrointestinal disorders** |  |
| Common | Nausea, diarrhoea, abdominal pain |
| **Musculoskeletal and connective tissue disorders** |  |
| Common | Arthralgia, myalgia |
| **General disorders and administration site conditions** |  |
| Very common | Fatigue |

**Laboratory abnormalities**

In clinical studies of SOF+DCV with or without ribavirin, 2% of patients had Grade 3 haemoglobin decreases; all of these patients received SOF+DCV+ribavirin. Grade 3/4 increases in total bilirubin were observed in 5% of patients (all in patients with HIV co-infection who were receiving concomitant atazanavir, with Child-Pugh A, B, or C cirrhosis, or who were post-liver transplant).

**HCV/HBV (hepatitis B virus) co-infection**

Cases of hepatitis B virus (HBV) reactivation, fatal on rare occasions, have been reported during or after treatment with direct-acting antiviral agents. HBsAg positive individulas are excluded from this study.

- - 1. **TREATMENT SCHEDULE**

The duration of SOF+DCV therapy will be determined by the Stratum the participant is in, and their response on therapy (see Figure 1). Note both SOF and DCV should always be co-administered.

- - 1. **DISPENSING**

The trial or clinic nurse will dispense SOF (and DCV) after taking the completed prescription form to the site pharmacy. All participants will receive a 28-day supply at Day 0. Further supplies will be provided at Day 28 dependent on response to therapy on Day 2 (mild disease) or Day 14 (cirrhosis). On no account should any drug assigned to a participant be used by anyone else. Unused drug must be returned to the site pharmacy immediately if a participant withdraws from treatment.

All drug dispensed at and returned to the site pharmacy should be documented on a site Accountability Log, maintained by a named person (trial pharmacist or research nurse). The designated trial pharmacist/nurse will, on receipt of supplies prior to the commencement of the trial, conduct an inventory and complete a receipt. Inventories will be conducted monthly, and logs returned to OUCRU CTU. Procedures for drug shipping, labelling, accountability, temperature monitoring and destruction will be detailed in the trial’s SOPs. OUCRU CTU will monitor drug accountability at site visits. There is no requirement for temperature monitoring of treatment packs after they are dispensed from pharmacy.

- - 1. **DRUG-DRUG INTERACTIONS**

SOF is a nucleotide prodrug, absorbed rapidly after oral administration, and cleaved into an active metabolite and inactive metabolite, GS-331007. SOF is a substrate of drug transporter P-gp and breast cancer resistance protein (BCRP) while GS-331007 is not. SOF and its metabolites can be subject to drug-drug interactions (see below) but are low risk as perpetrators of drug-drug interactions.

Medicinal products that are **potent P-gp inducers** in the intestine (i.e. rifampicin, rifabutin, St. John's wort (Hypericum perforatum), carbamazepine, phenobarbital and phenytoin) may significantly decrease SOF plasma concentration leading to reduced therapeutic effect of SOF. Co-administration with SOF is contraindicated.

Medicinal products that are **moderate P-gp inducers** in the intestine (e.g. oxcarbazepine and modafinil) may decrease SOF plasma concentration leading to reduced therapeutic effect. Co-administration with SOF is not recommended.

**Medicinal products that are P-gp and/or BCRP inhibitors**

Medicinal products that inhibit P-gp and/or BCRP can be used with SOF. See also www.hep-druginteractions.org.

- - 1. **DOSE MODIFICATIONS, INTERRUPTIONS & DISCONTINUATIONS**

**7.2.5.A Hepatic impairment**

No dose adjustment of SOF is required for participants with mild, moderate or severe hepatic impairment (Child Pugh [CP] class A, B or C).

**7.2.5.B Renal impairment**

No dose adjustment of SOF is required for participants with mild or moderate renal impairment. The safety of SOF has not been assessed in participants with severe renal impairment (estimated creatinine clearance <30 ml/min/1.73 m^2^) or end-stage renal disease requiring haemodialysis. These patients are excluded from the study.

**7.2.5.C Overdose**

No details are provided in the Summary of Product Characteristics (SPC); cases of overdose should therefore be discussed on a case-by-case basis with the trial investigators.

**7.2.5.D Stopping drug early**

If SOF has to be ceased for any reason then it is imperative the DCV is ceased too. Participants must not continue with DAA monotherapy.

**7.2.5.E Missed doses**

If a dose is missed and it is within 18 hours of the normal time, participants should be instructed to take the tablet as soon as possible and then participants should take the next dose at the usual time. If it is after 18 hours then participants should be instructed to wait and take the next dose at the usual time. Participants should be instructed **not to** take a double dose. Any doses missed during the treatment course should be taken at the end of the prescribed course.

- 1. DACLATASVIR (DCV)
     1. **PRODUCT**

The product is daclatasvir 60mg taken once a day, swallowed whole and not chewed (bitter taste). Although food does not affect the absorption of DCV, it will be taken with SOF, so they must both be taken with food.

**Side-effects:** see above Table.

- - 1. **TREATMENT SCHEDULE**

The duration of DCV therapy will be determined by the Stratum the participant is in, and their response on therapy (see study schema).

- - 1. **DISPENSING**

The trial or clinic nurse will dispense DCV (and SOF) after taking the completed prescription form to the site pharmacy. All participants will receive a 28-day supply at Day 0. Further supplies will provided at Day 28 dependent on response to therapy on Day 2 (mild disease) or Day 14 (cirrhosis). On no account should any drug assigned to a participant be used by anyone else. Unused drug must be returned to the site pharmacy immediately if a participant withdraws from treatment.

All drug dispensed at and returned to the site pharmacy should be documented on a site Accountability Log, maintained by a named person (trial pharmacist or research nurse). The designated trial pharmacist/nurse will, on receipt of supplies prior to the commencement of the trial, conduct an inventory and complete a receipt. Inventories will be conducted monthly, and logs returned to OUCRU CTU. Procedures for drug shipping, labelling, accountability, temperature monitoring and destruction will be detailed in the trial’s SOPs. OUCRU CTU will monitor drug accountability at site visits. There is no requirement for temperature monitoring of treatment packs after they are dispensed from pharmacy.

- - 1. **DRUG-DRUG INTERACTIONS**

DCV is a substrate of CYP3A4, P-gp and organic cation transporter (OCT). The dose of DCV may require alteration, particularly when taken in conjunction with other drugs which affect CYP3A4 metabolism (including ritonavir and azole drugs such as itraconazole). For updated information on drug-drug interactions see <http://www.hep-druginteractions.org>.

DCV (60mg) is supplied as 60mg tablets. Tablets will need to be split into two 30mg portions to provide dose adjustments for patients (in place of or in addition to 60mg tablets, depending on the interactions).

**Strong inducers of CYP3A4**

Administration of DCV is not recommended with strong inducers of CYP3A4. These active substances include but are not limited to phenytoin, carbamazepine, oxcarbazepine, phenobarbital, rifampicin, rifabutin, rifapentine, systemic dexamethasone, and the herbal product St John's wort *(Hypericum perforatum).*

**DCV as a perpetrator of drug-drug interactions**

DCV is an inhibitor of P-gp, organic anion transporting polypeptide (OATP) 1B1, OCT1 and breast cancer resistance protein (BCRP). Administration of DCV may increase systemic exposure to medicinal products that are substrates of P-gp, OATP 1B1, OCT1 or BCRP, which could increase or prolong their therapeutic effect and adverse reactions. Caution should be used if the medicinal product has a narrow therapeutic range e.g. Dabigatran etexilate. DCV is a very weak inducer of CYP3A4 and dose adjustment of concomitantly administered CYP3A4 substrates is not necessary.

**7.3.5 Dose Modifications, Interruptions & Discontinuations**

**7.3.5.A Hepatic impairment**

No dose adjustment of DCV is required for participants with mild (Child-Pugh A, score 5-6), moderate (Child-Pugh B, score 7-9) or severe (Child-Pugh C, score ≥10) hepatic impairment.

**7.3.5.B Renal impairment**

No dose adjustment of DCV is required for participants with any degree of renal impairment.

**7.3.5.C Bradyarrhythmia**

Cases of severe bradycardia and heart block have been observed when SOF+DCV is taken with concomitant amiodarone with or without other drugs that lower heart rate. The mechanism is not established. Therefore, individuals taking amiodarone or other anti-arrhythmics (including those with permanent pacemakers) are not eligible for participation in this study. If amiodarone is required during the course of the study, SOF+DCV should be ceased immediately, and the participant closely monitored “on study, but off study drug”.

**7.3.5.D Interactions with other common medications**

Patients currently receiving medication known to interact with study medications for which dose adjustment for daclatasvir or sofosbuvir would be recommended in the Summary of Product Characteristics are not eligible for the trial. See <https://www.hep-druginteractions.org/> for a summary.

**7.3.5.E Overdose**

There is limited experience of accidental overdose of DCV in clinical studies. In phase 1 clinical studies, healthy subjects who received up to 100 mg once daily for up to 14 days or single doses up to 200 mg had no unexpected adverse reactions. There is no known antidote for overdose of DCV. Treatment of overdose with DCV should consist of general supportive measures, including monitoring of vital signs, and observation of the patient's clinical status. Because DCV is highly protein bound (99%) and has a molecular weight >500, dialysis is unlikely to significantly reduce plasma concentrations.

**7.3.5.F Stopping drug early**

If DCV has to be ceased for any reason then it is imperative the SOF is ceased too. Participants must not continue with DAA monotherapy.

**7.3.5.G Missed doses**

If a dose is missed and it is within 18 hours of the normal time, participants should be instructed to take the tablet as soon as possible and then participants should take the next dose at the usual time. If it is after 18 hours then participants should be instructed to wait and take the next dose at the usual time. Participants should be instructed **not to** take a double dose. Any doses missed during the treatment course should be taken at the end of the prescribed course.

- 1. Blinding

Not applicable, the study is open-label.

- 1. Overdose of Trial Medication

See above under each individual study drug.

- 1. Protocol Treatment Discontinuation

See above under each individual drug. Both drugs must be discontinued simultaneously to avoid receiving DAA monotherapy.

**Pregnancy**

Pregnancy is an exclusion criterion for participation. DCV should not be used during pregnancy or in women of childbearing potential not using contraception. The potential risk to a human foetus is unknown, because there are no data from the use of DCV in pregnant women. However, studies of DCV in animals have shown embryotoxic and teratogenic effects. As pregnancy tests are performed repeatedly during the study, foetal exposure to study drugs will be limited. Furthermore, use of highly effective contraception is recommended throughout the trial and for 5 weeks after completion of DCV therapy. Any participant who becomes pregnant on study must be counselled appropriately on the risks of DAA exposure to the unborn child, taking into account the duration of DAAs received.

- 1. Adherence

As the intervention will start immediately following enrolment, suitable participant information and fully informed consent procedures will ensure that participants understand the trial requirements. Therefore, any non-adherence will likely be a consequence of the intervention itself (e.g. drug intolerance or toxicity) which would also be likely to occur if it were incorporated within clinical practice, i.e. non-adherence will likely be part of the strategy being evaluated. The intention-to-treat analysis will therefore incorporate the level of non-adherence as would be anticipated in general clinical practice. Non-adherence will be assessed using participant self-report and pill counts.

- 1. Treatment Data Collection

See Trial Assessment Schedule

- 1. Non-trial Treatment

As this is a pragmatic trial, decisions regarding all other medications taken by the participant will be determined by the attending physician according to the clinical circumstances.

- - 1. **Treatment After end of study drug**

The treatment of participants after the study finishes should be determined by the local physician responsible for care and in line with national guidance. Normal standards of clinical care should be followed with careful documentation of their management in the CRF.

1. Safety Reporting

The principles of ICH GCP require that both investigators and sponsors follow specific procedures when notifying and reporting adverse events or reactions in clinical trials. These procedures are described in this section of the protocol. [Section 8.1 - Definitions](#Definitions) lists definitions, [Section 8.2 – Investigator Responsabilites](#InvestigatorResponsibilities) gives details of the investigator responsibilities*.*

- 1. Definitions

The principles of ICH GCP apply to this trial protocol, as per Table 1: Definitions below.

*Table 1: Definitions*

| *Table* | *Definition* |
| --- | --- |
| Adverse Event (AE) | Any untoward medical occurrence in a participant or clinical trial subject to whom an investigational medicinal product has been administered including occurrences that are not necessarily caused by or related to that product. |
| Adverse Reaction (AR) | Any untoward and unintended response to an investigational medicinal product related to any dose administered. |
| Unexpected Adverse Reaction (UAR) | An adverse reaction, the nature or severity of which is not consistent with the information about the investigational medicinal product in question set out in the Summary of Product Characteristics (SPC) for that product. |
| Serious Adverse Event (SAE) or Serious Adverse Reaction (SAR) or Suspected Unexpected Serious Adverse Reaction (SUSAR) | Respectively any adverse event, adverse reaction or unexpected adverse reaction that:   - Results in death - Is life-threatening* - Requires hospitalisation or prolongation of existing hospitalisation** - Results in persistent or significant disability or incapacity - Consists of a congenital anomaly or birth defect - Is another important medical condition*** |

*The term life-threatening in the definition of a serious event refers to an event in which the participant is at risk of death at the time of the event; it does not refer to an event that hypothetically might cause death if it were more severe, for example, a silent myocardial infarction.

**Hospitalisation is defined as an inparticipant admission, regardless of length of stay, even if the hospitalisation is a precautionary measure for continued observation. Hospitalisations for a pre-existing condition (including elective procedures that have not worsened) do not constitute an SAE.

*** Medical judgement should be exercised in deciding whether an AE or AR is serious in other situations. The following should also be considered serious: important AEs or ARs that are not immediately life-threatening or do not result in death or hospitalisation but may jeopardise the subject or may require intervention to prevent one of the other outcomes listed in the definition above; for example, a secondary malignancy, an allergic bronchospasm requiring intensive emergency treatment, seizures or blood dyscrasias that do not result in hospitalisation or development of drug dependency*.*

Adverse Events include:

- An exacerbation of a pre-existing illness
- An increase in frequency or intensity of a pre-existing episodic event or condition
- A condition (even though it may have been present prior to the start of the trial) detected after trial drug administration
- Continuous persistent disease or a symptom present at baseline that worsens following administration of the study treatment
  - 1. **Medicinal Products**

An investigational medicinal product is defined as the tested investigational medicinal product (IMP) and the comparators used in the study. This therefore includes:

- daclatasvir
- sofosbuvir

Adverse reactions include any untoward or unintended response to drugs. Reactions to an IMP or comparator should be reported appropriately.

- - 1. **Exempted Adverse Events**

In the context of this trial Adverse Events **do not include**:

- Medical or surgical procedures; the condition that leads to the procedure is the adverse event
- Pre-existing disease or a condition present before treatment that does not worsen
- Hospitalisations where no untoward or unintended response has occurred, e.g. elective cosmetic surgery, social admissions
- Overdose of medication without signs or symptoms
- Disease-related events that are not fatal
  1. Investigator Responsibilities

All clinical grade 3 or 4 AEs, serious AEs, and all ARs, whether expected or not, should be recorded in the CRF. Non-serious grade 1 or 2 AEs and laboratory grade 3 or 4 AEs are only reported if they lead to a change or interruption in treatment (SOF, DCV or any other medication being taken by the participant).

A laboratory abnormality must be recorded as a clinical adverse event only if it is associated with an intervention. Intervention includes, but is not limited to, discontinuation of a current treatment, dose reduction/delay of a current treatment, or initiation of a specific treatment. In addition, any medically important laboratory abnormality may be reported as an adverse event at the discretion of the investigator. This would include a laboratory result for which no intervention is needed, but the abnormal value suggests a disease or organ toxicity. Laboratory events will be graded according to Common Toxicity Criteria for Adverse Events (CTCAE) definitions.

SAEs and SARs should be notified to the OUCRU CTU immediately and within no more than 24 hours of the investigator becoming aware of the event.

- - 1. **Investigator Assessment**
       1. Seriousness

When an AE or AR occurs, the investigator responsible for the care of the participant must first assess whether or not the event is serious using the definition given in [Table 1: Definitions.](#Table8_1Definitions) If the event **is serious** or is fatal, then an SAE Form must be completed and the OUCRU CTU notified within 24 hours.

- - - 1. Severity or Grading of Adverse Events

The severity of all AEs and/or ARs (serious and non-serious) in this trial should be graded using the Common Toxicity Criteria for Adverse Events gradings (Appendix III - Toxicity Gradings and Management).

- - - 1. Causality

The investigator must assess the causality of all serious events or reactions in relation to the trial therapy using the definitions in [Table 2: Assigning Type of SAE Through Causality.](#Table7_2AssigningTypeOfSAE) There are five categories: unrelated, unlikely, possible, probable, and definitely related. If the causality assessment is unrelated or unlikely to be related, the event is classified as an SAE. If the causality is assessed as possible, probable or definitely related, then the event is classified as an SAR.

Table 2 Assigning Type of SAE Through Causality

| *Relationship* | *Description* | *SAE Type* |
| --- | --- | --- |
| Unrelated | There is no evidence of any causal relationship | Unrelated SAE |
| Unlikely | There is little evidence to suggest that there is a causal relationship (for example, the event did not occur within a reasonable time after administration of the trial medication). There is another reasonable explanation for the event (for example, the participant’s clinical condition, other concomitant treatment). | Unrelated SAE |
| Possible | There is some evidence to suggest a causal relationship (for example, because the event occurs within a reasonable time after administration of the trial medication). However, the influence of other factors may have contributed to the event (for example, the participant’s clinical condition, other concomitant treatments). | SAR |
| Probable | There is evidence to suggest a causal relationship and the influence of other factors is unlikely. | SAR |
| Definitely | There is clear evidence to suggest a causal relationship and other possible contributing factors can be ruled out. | SAR |

- - - 1. Expectedness

If there is at least a possible involvement of the trial treatment, the investigator must assess the expectedness of the event. An unexpected adverse reaction is one not previously reported in the current Summary of Product Characteristics (SPC) at the time the event occurred, or one that is more frequent or more severe than previously reported. The definition of an unexpected adverse reaction (UAR) is given in [Table 1: Definitions.](#Table8_1Definitions) If a SAR is assessed as being unexpected, it becomes a SUSAR.

Investigators should always check the current version of the SPC.

- - - 1. Notification

The OUCRU CTU should be notified of all SAEs immediately and within no more than 24 hours of the investigator/study team becoming aware of the event. Investigators should notify the OUCRU CTU of all SAEs occurring from the time of enrolment until the participant finishes their 12 week follow-up. SARs and SUSARs must be notified to the OUCRU CTU until trial closure. CTU will perform an initial check of the report, request any additional information, and ensure it is reviewed on a weekly basis. The causality assessment given by the local investigator at the hospital cannot be overruled; in the case of disagreement, both opinions will be provided in any subsequent reports. All SAE information must be recorded on an SAE form and sent or emailed, to the OUCRU CTU. Additional and further requested information (follow-up or corrections to the original case) will be detailed on a new SAE Report Form and sent to CTU.

- - 1. **Notification Procedure**

The OUCRU CTU is undertaking the duties of trial sponsor with regard to safety reporting and is responsible for the reporting of SUSARs and other SARs to the regulatory authorities and the research ethics committees, as appropriate.

Safety reporting will focus on events of potential relevance to the trial intervention. The following events will be reported to the relevant authorities:

- All unexpected serious adverse events
- All serious adverse events judged to be definitely, probably or possibly related to the trial interventions (SOF+DCV)
- All deaths

In Vietnam, the above SAEs will be reported as soon as possible and within 2 working days of the time of acknowledgement of the SAE to the site IRB. An initial written report will be sent as soon as possible and not later than 7 days from the acknowledgement of the event to the Vietnam MoH ethics committee (EC). The format and content of the initial report should follow the Vietnam MoH EC report template and include all information available at the time of reporting. A follow up report with complete details will be sent within 15 days since the time of acknowledgement if the initial report does not contain the details of event resolution. All foreseeable and predefined SAEs will not be reported immediately but will be included in the annual report to the VN MoH EC.

1. Statistical Considerations
   1. Sample Size justification

For both treatment strata, the target cure rate is 90%, with an unacceptably low cure rate of 70% (final lower 95% CI limit should exceed this). Assuming 90% power and one-sided alpha=0.05, requires 37 participants to exclude the null hypothesis (cure <90%). Assuming 5% loss to follow-up in both strata, and that 65% (based on [[12](#_ENREF_12)]) and 90% achieve on-treatment response and receive shortened therapy in Stratum A and B respectively, 60 participants would be required in Stratum A (pooling genotypes), and 43 in Stratum B. 5% loss follow-up is consistent with experience in the local setting which has generally followed patients for much longer than 12 weeks post end of treatment as here [personal communication, OUCRU].

- 1. Analysis Plan (Brief)

The analyses will be described in detail in a full Statistical Analysis Plan. This section summarises the main issues.

The primary analysis would be intention-to-treat, including all participants regardless of treatment received. Primary analysis of SVR12 (the primary endpoint) would be as observed (those not meeting the formal “failure” definition but discontinuing first-line treatment early and not undergoing re-treatment with detectable HCV RNA 12 weeks later counted as not achieving the primary endpoint). Any missing data at 12 weeks post EOT due to assay failure or missed visit would be multiply imputed within stratum based on all viral load measurements and baseline characteristics, using chained estimating equations. Continuous variables would be transformed for normality, and data augmented following standard recommendations to avoid problems with perfect prediction. The distribution of imputed data would be checked versus observed data.

Secondary analyses for each outcome would be based on a per-protocol population, which we (arbitrarily) define as receiving >90% and <110% of the prescribed duration of first-line treatment. Assessment of treatment received will be based on both prescription and self-reported adherence.

A further secondary analysis will exclude reinfections leading to rebound virus, with reinfection determined by genotype and full genome sequencing.

As the duration of follow-up is relatively short, primary analysis would consider outcomes as binary. Proportions and their 95% CI would be estimated using Poisson regression.

1. Data management
   1. source data

Source documents are where data are first recorded, and from which participants’ CRF data are obtained. These include, but are not limited to, hospital records (from which medical history and previous and concurrent medication may be summarised into the CRF), clinical and office charts, laboratory and pharmacy records, and correspondence. CRF entries will be considered source data if the CRF is the site of the original recording (e.g. there is no other written or electronic record of data). All documents will be stored safely in confidential conditions. On all trial-specific documents, other than the signed consent, the participant will be referred to by the trial participant number/code, not by name.

- 1. Direct Access to Participant Records

Participating investigators should agree to allow trial-related monitoring, including audits, ethics committee review and regulatory inspections by providing direct access to source data and documents as required. Participants’ consent for this must be obtained. Such information will be treated as strictly confidential and will in no circumstances be made publicly available.

The following data should be verifiable from source documents:

- all signed consent forms
- dates of assessments including dates specimens were taken and processed in the laboratory
- eligibility and baseline values for all participants
- all clinical endpoints
- all serious adverse events and grade 3 or 4 clinical adverse events
- routine participant clinical and laboratory data
- drug compliance
- dates drug dispensed and (if necessary) drugs returned
- pharmacy/clinic drug logs
- concomitant medication.
  1. Data collection and management responsibilities

Data collection is the responsibility of the clinical trial staff at the site under the supervision of the site PI. The PI is responsible for ensuring the accuracy, completeness, legibility, and timeliness of the data reported.

Clinical data (including AEs, concomitant medications, and expected adverse reactions data) and clinical laboratory data will be entered into CliRes, a Title 21 Code of Federal Regulations (CFR) Part 11-compliant data capture system provided by the OUCRU IT department. The data system includes password protection and internal quality checks, such as automatic range checks, to identify data that appear inconsistent, incomplete, or inaccurate. Clinical data will be entered directly from the source documents.

- 1. Data Recording and Record Keeping

All trial data will be recorded on paper CRFs and entered into CliRes. The participants will be identified by a unique trial specific code. Names other identifying details will NOT be included in any trial data electronic files.

- 1. trial Records retention

CRFs, and administrative documentation will be kept in a secure location and held for 15 years after the end of the trial so it can be available for future reference including audit. Clinical information will not be released without written permission, except as necessary for monitoring, auditing and inspection purposes. During this period, all data should be accessible to the competent authorities with suitable notice. Electronic data will be kept for at least 20 years at the OUCRU CTU.

- 1. protocol deviations and violations

A protocol deviation is any non-compliance with the clinical trial protocol or GCP requirements. If such a deviation results in an impact on patient safety or scientific integrity it becomes a protocol violation. The non-compliance may be either on the part of the participant, the investigator, or the study site staff. Whenever deviations occur, corrective actions are to be developed by the site and implemented promptly.

It is the responsibility of the site investigators to use continuous vigilance to identify and report protocol deviations and violations. All deviations and violations must be documented in source documents and reported to the OUCRU CTU within 2 days of being identified. In addition, protocol violations must be reported to the relevant ethics committees.

- 1. publication and data sharing policy

All publications are to be approved by the trial management group (TMG) before submission for publication.

The TMG will maintain a list of investigators to be presented in an appendix at the end of the paper. This list will include investigators who contributed to the investigation being reported but who are not members of the writing committee. In principle, substudy reports should include all investigators for the main study, although in some instances where a smaller number of investigators have made any form of contribution, it may be appropriate to abbreviate the listing. All headline authors in any publication arising from the main study or sub-studies must have made a substantive academic or project management contribution to the work that is being presented. “Substantive” must be defined by a written declaration of exactly what the contribution of any individual is believed to have been. In addition to fulfilling the criteria based on contribution, additional features that will be considered in selecting an authorship group will include the recruitment of participants who contributed data to any set of analyses contained in the manuscript and/or the conduct of analyses (laboratory and statistical), leadership and coordination of the project in the absence of a clear academic contribution.

In line with the Wellcome and MRC Policy and Guidance on Sharing of Research Data from Population and Patient Studies, results of publicly-funded research should be freely available, manuscripts arising from the trial will, wherever possible, be submitted to peer-reviewed journals which enable Open Access via UK PubMed Central (PMC) within six months of the official date of final publication. All publications will acknowledge the trial's funding sources.

In line with research transparency and greater access to data from trials all of OUCRU’s clinical trials are registered with the ISRCTN registry and a data sharing policy is in place. This trial will follow the OUCRU data sharing policy, which is based on a controlled access approach with a restriction on data release that would compromise an ongoing trial or study. Data exchange complies with Information Governance and Data Security Policies in all of the relevant countries.

1. Quality Assurance & Control
   1. Risk Assessment

The Quality Assurance (QA) and Quality Control (QC) considerations have been based on a formal Risk Assessment, which acknowledges the risks associated with the conduct of the trial and how to address them with QA and QC processes. QA includes all the planned and systematic actions established to ensure the trial is performed and data generated, documented and/or recorded and reported in compliance with the principles of ICH GCP and applicable regulatory requirements. QC includes the operational techniques and activities done within the QA system to verify that the requirements for quality of the trial-related activities are fulfilled.

- 1. Central Monitoring at OUCRU CTU

Data will be collected on paper CRFs will be entered and stored on a central database in OUCRU. The database will be checked at OUCRU CTU for missing or unusual values (range checks) and checked for consistency within participants over time. If any such problems are identified, the site will be contacted and asked to verify or correct the data. OUCRU CTU will also send reminders for any overdue and/or missing data with the regular inconsistency reports of errors.

Other essential trial issues, events and outputs will be detailed in the Data Management, Monitoring and Quality Management Plans that are based on the trial-specific Risk Assessment.

- 1. On-site Monitoring

A site initiation visit will be conducted by staff from the OUCRU CTU. All essential site staff including the PI, lead pharmacist and lead research nurse must be in attendance. The initiation training will include training in the administration of study drug, as well as the trial procedures. Monitoring will then be carried out by OUCRU CTU staff. The frequency, type and intensity of routine monitoring and the requirements for triggered monitoring will be detailed in the Monitoring Plan which will also detail the procedures for review and sign-off. The monitoring will adhere to the principles of ICH GCP and the Monitoring Plan.

The monitors will require access to all participant medical records including, but not limited to, laboratory test results and prescriptions. The investigator (or delegated deputy) should work with the monitor to ensure that any problems detected are resolved.

1. Ancillary Studies

We will conduct two ancillary studies within the trial. The results of these ancillary studies will be reported separately from the results of the main trial. The studies are as follows:

- 1. Pharmacokinetics

Venous plasma samples will be collected for sofosbuvir, GS-331007, and daclatasvir for pharmacokinetic evaluations.

Frequent pharmacokinetic samples will be collected in 40 patients with mild liver disease. Five samples will be collected in each patient after the first dose and at day 28, resulting in a total of 10 samples per patient. Samples will be collected according to sampling schedule A or B after the first dose and schedule B or A, respectively, at day 28. All samples can be collected through an indwelling venous catheter, depending on patient preference.

Sampling schedule A: 0.5, 2, 4, 6, 24 hours after dose

Sampling schedule B: 1, 3, 5, 8, 24 hours after dose

Sparse pharmacokinetic samples will be collected in all 103 patients. A total of three samples per patient will be collected randomly at week D7, D14 and end-of-treatment (either D28 or D56). All three samples will be collected between 0-8 hours after dose through venepuncture.

This sampling schedule will result in a maximum of 13 samples in 40 patients with mild disease and 3 samples in 63 patients. One mL of blood will be collected at each sampling occasion resulting in a maximum volume of 13 mL of blood per patient for pharmacokinetic evaluations. All samples will be shipped on dry ice to the Department of Clinical Pharmacology, Mahidol Oxford Tropical Medicine Research Unit, Bangkok, Thailand for drug measurements. Drug quantification will be performed using liquid chromatography tandem mass-spectrometer.

Dense pharmacokinetic samples collected at day 0 (enrolment) and day 28 will be analysed using an individual model-independent approach (i.e. non-compartmental analysis), to characterise individual pharmacokinetic parameters and drug exposures in patients with mild disease. All samples collected during the study will also be pooled and analysed using nonlinear mixed-effects modelling to develop a detailed pharmacokinetic model that describes the concentration-time relationship and possible impact of clinical covariates, such as age or body weight. The relationship between individual drug concentrations and dynamic viral measurements will be evaluated by nonlinear mixed-effect modelling (i.e. sequential incorporation of the pharmacokinetic model structure and E_MAX_-type pharmacodynamic model).

- 1. Health Economics

Understanding the cost and cost-effectiveness interventions is a vital part of developing public health policy. Although there have been several studies investigating the cost-effectiveness of HCV treatment, these have nearly exclusively focused on high-income countries. Due to this, there are currently almost no studies investigating the cost or cost-effectiveness of using generic drugs for HCV treatment in LMIC, where there is a huge untreated burden.

**Costing of the HCV treatment:**

We will develop and pilot cost data collection tools that will allow us to perform a micro-costing of the investigated HCV treatment strategies. This method involves costing every input consumed in the treatment of a particular patient. Within our costing approach, we will also collect the costs incurred by patients and their families (such as their travel and food expenses). Using this wider, “societal”, perspective is becoming increasingly recognized as the gold standard for economic evaluations. This cost data will allow us to understand the costs of HCV treatment in Vietnam (for both the health system and the patients). This is also important for understanding potential barriers for receiving treatment.

The data will be collected based on the Hospital for Tropical Diseases electronic billing system, with additional questionnaires administered to the patients. It will be necessary to ask the patients and the informal caregivers basic demographical information (including questions regarding their salary and employment status), and the costs they incurred to travel to the clinic. This data will be collected using an outpatient costing questionnaire. This will be given to 20 patients from Stratum A and 20 from Stratum B. It will be administered three times to each patient (Stratum A timepoints: (D0, EOT (28/56), D84), Stratum B timepoints: (D0, D84, Week 12)). The patients will be selected randomly (and will not be given the economic burden questionnaire – to avoid repetition).

Based on the responses and data collected we will make improvements to the outpatient costing questionnaire. The finalized questionnaire will be used on a larger number of patients in the up-coming Wellcome trust HCV study. This will allow detailed and accurate cost data to be collected in a standardised manner which will be used to perform cost-effectiveness of different treatment strategies. An advantage of using this more detailed micro-costing approach is that it will allow us to investigate how the costs of providing HCV treatment could change if the coverage was scaled up, or if it was integrated into another control programme (such as those for HIV). This will help us to understand how generalizable our findings are to other low and middle-income settings.

**Effectiveness of the HCV treatment**

Using established models describing the progressive stages of chronic HCV, we will aim to translate the outcomes of the trial into an estimated number of disability-adjusted life years (DALYs) averted. This will follow the approach used within the 2010 global burden of disease study. In addition, we will aim to administer the EQ5D-3L quality of life questionnaire to all of the enrolled patients at baseline (before treatment), midway during treatment, and after treatment (at the EOT+12 week visit). This data will allow us to measure the number of quality-adjusted life-years (QALY) saved due to the therapy.

1. Regulatory & Ethical considerations

All regulatory requirements (including safety reporting, see Section 8 - Safety Reporting and below) will be met by the sponsor or their delegated authorities.

- 1. Compliance

The trial will end at the final first-line or re-treatment EOT+12 week visit of all enrolled participants. Because the trial may contain a re-treatment phase, this may not necessarily be the final EOT+12 week visit of the last enrolled patient.

- - 1. **Regulatory Compliance**

The trial complies with the principles of the Declaration of Helsinki (2008) and will be conducted in compliance with the approved protocol and the principles of Good Clinical Practice (GCP).

- - 1. **SITE COMPLIANCE**

All sites will comply with the above. An agreement will be in place between the site and OUCRU, setting out respective roles and responsibilities.

The site will inform the OUCRU CTU as soon as they are aware of a possible serious breach of compliance. For the purposes of this regulation, a 'serious breach' is one that is likely to affect to a significant degree:

- The safety or physical or mental integrity of the subjects in the trial, or
- The scientific value of the trial
  1. Ethical Conduct of the Study
     1. **Ethical Considerations**

The primary benefit of participation for individual patients in this trial is that >90% of individuals will achieve cure of hepatitis C without interferon-based treatment within the protocol. For many, interferon will have been a barrier to accessing and/or adhering to treatment previously. Barriers to accessing treatment are likely to remain even with increasing availability of directly acting antivirals (DAAs), as demand for treatment is expected to exceed the capacity to deliver it in setting such as Vietnam. The primary outcome measure (sustained virological response 12 weeks after end of treatment, SVR12) has been associated with significant reductions in all-cause mortality, cirrhosis and liver cancer [[18](#_ENREF_18)], and so achieving cure will significantly reduce individual patient’s future risks of these events. The study will use licensed medications that have been recommended as part of the WHO Essential Medicines list since 2015.

For the majority of participants, it is expected that cure can be achieved with a shorter duration of therapy than they would have received through routine care. The main risk in participation is that short-course therapy will not lead to SVR12/cure and there is a theoretical possibility that participants may develop viral resistance perhaps compromising future treatment options. However, emerging data in this area [[15](#_ENREF_15)] [[23](#_ENREF_23), [24](#_ENREF_24)] are reassuring in that, even with emergence of NS5A mutations, cure rates of >90% can be achieved with retreatment. On this basis approval has been given to previous studies of shortened treatment courses (e.g. STOPHCV-1). As recommended in a recent review, all those not cured initally will be routinely retreated for 12 weeks [[25](#_ENREF_25)]. Even assuming conservative estimates of retreatment success, >90% of those included in study should achieve SVR12.

The intervention agents used within this trial have already completed Phase III trials (ALLY studies) and have been approved for use by regulatory bodies including FDA, EMA, NICE and WHO. There are data on adverse event profiles from over 300 individuals with daclatasvir [[26](#_ENREF_26)] and >10,000 participants treated with sofosbuvir [[27](#_ENREF_27)]. Of the intervention treatments in this study and no major safety/toxicity concerns have yet emerged; as such, given the future mortality and morbidity, the potential for cure outweighs the risks of treatment for individuals. Because these medications are now being rolled out more widely, it is possible that new evidence of toxicity will emerge, but given that treatment has a finite duration, and the trial will test response guided shortened courses, the likelihood of new toxicities emerging to place individual participants at unacceptable risk is low.

Societal benefits arising from understanding which individuals can be cured with shortened therapy are potentially substantial. The treatment interventions within this study are currently priced at a level where access will not be affordable for all infected participants, many of whom are likely to need to self-fund treatment, at least in part. The findings from this study will inform how health services can efficiently provide treatment to those with mild disease, avoid over-treating many participants and allow more participants to be cured within a finite resource. Furthermore, the findings will inform the more precise use of new treatments in participants for whom a shortened 12 week course of therapy will still be challenging and will inform strategies that will allow individuals who may be cured with as little as 4 weeks of treatment to be identified.

- - 1. **Ethical Approvals**

The trial will be approved by the Imperial Research Ethics Committee, Oxford Tropical Research Ethics Committee, the Ethics Committee of the Hospital for Tropical Diseases and the Vietnam Ministry of Health. Regulatory approval will be given by the Drug Administration of Vietnam (DAV).

Any amendments will be submitted and approved by these ethics committees.

- - 1. **Confidentiality**

The investigator must assure that participants’ anonymity will be maintained and that their identities are protected from unauthorised parties. Participants will be assigned a trial identification number and this will be used on CRFs; participants will not be identified by their name. The investigator will keep securely a participant trial register showing identification numbers, surnames and date of birth. This unique trial number will identify all laboratory specimens, case record forms, and other records and no names will be used, in order to maintain confidentiality.

- - 1. **Expenses**

The study funding will cover the following costs:

- Study specific screening tests and procedures.
- Study related diagnostic, treatment and hospital costs from enrolment to the final study visit at EOT+12 weeks (including re-treatment for any participants in the mild disease cohort failing first-line treatment).
- Hospital costs (including costs of hospital accomodation, related investigations, and treatment), for participants admitted for adverse events
- Treatment of any adverse events which are caused by study participation.
- Travel expenses for the patient to attend follow-up visits (based on actual costs at standard OUCRU rates).

The details of the costs covered by the trial will be set out in the contract between OUCRU and the study site, but will follow the principles described above.

1. Oversight & Trial Committees
   1. Trial Management GROUP (TMG)

A Trial Management Group (TMG) will be formed to conduct the day-to-day management of the trial at the OUCRU CTU. This will include the Chief Investigator, Site PI, Trial Statistician, Clinical Project Manager, Trial Manager, Lab Manager and Data Manager. The group will meet at least once per month, although may meet more or less often as required. The group will discuss issues related to the progress of the trial at the site and to ensure that the trial is running well.

The full details can be found in the TMG Charter.

- 1. Data Monitoring Committee (DMC)

An independent Data Monitoring Committee (DMC) will oversee the safety of the trial. The DMC will review the confidential, accumulating data for the trial separately by treatment group. A DMC Charter will be drawn up that describes the membership of the DMC, relationships with other committees, terms of reference, decision-making processes, and the approximate timing and frequency of any interim analyses.

See Appendix I - Data Monitoring Committee Membership for membership.

1. Finance and insurance

15.1 Funding

The trial is supported by an award from the UK MRC (SEARCH) as part of the Global Challenge Research Fund calls GCRF FA 260.

15.2 INSURANCE

The conduct of this study is funded by the MRC (UK) and sponsored by the Imperial College London. The University has a specialist insurance policy in place.

1. Conflict of Interest

The independence of this study from any actual or perceived influence, such as by the pharmaceutical industry, is critical. Therefore, any actual conflict of interest of persons who have a role in the design, conduct, analysis, publication, or any aspect of this trial will be disclosed and managed. Furthermore, persons who have a perceived conflict of interest will be required to have such conflicts managed in a way that is appropriate to their participation in the trial. The study leadership has established policies and procedures for all study group members to disclose all conflicts of interest.

1. Protocol Amendments
2. References

1. Stanaway, J.D., et al., *The global burden of viral hepatitis from 1990 to 2013: findings from the Global Burden of Disease Study 2013.* Lancet, 2016. **388**(10049): p. 1081-8.

2. Gower, E., et al., *Global epidemiology and genotype distribution of the hepatitis C virus infection.* J Hepatol, 2014. **61**(1 Suppl): p. S45-57.

3. Messina, J.P., et al., *Global distribution and prevalence of hepatitis C virus genotypes.* Hepatology, 2015. **61**(1): p. 77-87.

4. Pybus, O.G., et al., *Genetic history of hepatitis C virus in East Asia.* J Virol, 2009. **83**(2): p. 1071-82.

5. Rehermann, B., *HCV in 2015: Advances in hepatitis C research and treatment.* Nat Rev Gastroenterol Hepatol, 2016. **13**(2): p. 70-2.

6. Sulkowski, M.S., et al., *Daclatasvir plus sofosbuvir for previously treated or untreated chronic HCV infection.* N Engl J Med, 2014. **370**(3): p. 211-21.

7. Poordad, F., et al., *Daclatasvir with sofosbuvir and ribavirin for hepatitis C virus infection with advanced cirrhosis or post-liver transplantation recurrence.* Hepatology, 2016. **63**(5): p. 1493-505.

8. *EASL Clinical Practice Guidelines: Management of hepatitis C virus infection.* 2016.

9. Sann, K., *Real-world effectiveness and safety of Daclatasvir/Sofosbuvir with or without Ribavirin among genotype 5 and 6 Hepatitis C Virus patients*, in *AASLD Liver Learning*. 2016.

10. Wyles, D.L., et al., *Daclatasvir plus Sofosbuvir for HCV in Patients Coinfected with HIV-1.* N Engl J Med, 2015. **373**(8): p. 714-25.

11. El-Shabrawi, M.H., et al., *Shortened 8 Weeks Course of Dual Sofosbuvir/Daclatasvir Therapy in Adolescent Patients, With Chronic Hepatitis C Infection.* J Pediatr Gastroenterol Nutr, 2018. **66**(3): p. 425-427.

12. Lau, G., et al., *Effi cacy and safety of 3-week response-guided triple direct-acting antiviral therapy for chronic hepatitis C infection: a phase 2, open-label, proof-of-concept study.* Lancet Gastroenterology and Hepatology, 2016. **1**(2): p. 97-104.

13. Lawitz, E., et al., *Retreatment of patients who failed 8 or 12 weeks of ledipasvir/sofosbuvir based regimens with ledipasvir/sofosbuvir for 24 weeks*, in *ILC 2015*. 2015: Vienna.

14. Kohli, A., et al., *Four-Week Direct-Acting Antiviral Regimens in Noncirrhotic Patients With Hepatitis C Virus Genotype 1 Infection: An Open-Label, Nonrandomized Trial.* Ann Intern Med, 2015. **163**(12): p. 899-907.

15. Wilson, E.M., et al., *Successful Retreatment of Chronic HCV Genotype-1 Infection With Ledipasvir and Sofosbuvir After Initial Short Course Therapy With Direct-Acting Antiviral Regimens.* Clin Infect Dis, 2016. **62**(3): p. 280-8.

16. Kohli, A., et al., *Virological response after 6 week triple-drug regimens for hepatitis C: a proof-of-concept phase 2A cohort study.* Lancet, 2015. **385**(9973): p. 1107-13.

17. Wilson, E., et al., *High Efficacy of retreatment with LDV/SOF in HCV Patients who failed initial short course therapy with combination DAA regimens (NIH SYNERGY trial)* in *ILC 2015*. 2015: Vienna.

18. Simmons, B., et al., *Long-Term Treatment Outcomes of Patients Infected With Hepatitis C Virus: A Systematic Review and Meta-analysis of the Survival Benefit of Achieving a Sustained Virological Response.* Clin Infect Dis, 2015. **61**(5): p. 730-40.

19. Nitta, Y., et al., *Liver stiffness measured by transient elastography correlates with fibrosis area in liver biopsy in patients with chronic hepatitis C.* Hepatol Res, 2009. **39**(7): p. 675-84.

20. Menezes, E.G., et al., *Strategies for serum chemokine/cytokine assessment as biomarkers of therapeutic response in HCV patients as a prototype to monitor immunotherapy of infectious diseases.* Antiviral Res, 2017. **141**: p. 19-28.

21. Neesgaard, B., M. Ruhwald, and N. Weis, *Inducible protein-10 as a predictive marker of antiviral hepatitis C treatment: A systematic review.* World J Hepatol, 2017. **9**(14): p. 677-688.

22. Omar, H., et al., *Generic daclatasvir plus sofosbuvir, with or without ribavirin, in treatment of chronic hepatitis C: real-world results from 18 378 patients in Egypt.* Aliment Pharmacol Ther, 2018. **47**(3): p. 421-431.

23. Poordad, F., et al., C-Swift: *Grazoprevir/Elbasvir + Sofosbuvir in Noncirrhotic, treatment-naïve patients with Hepatitis C virus Genotype 1 infection, for duration of 4, 6 or 8 weeks and genotype 3 infection for durations of 8 or 12 weeks in EASL.2015*: Vienna, Austria.

24. Poordad, F., et al., *C-SWIFT Retreatment (Part B): 12 Weeks of Elbasvir/Grazoprevir with Sofosbuvir and Ribavirin Successfully Treated G1-Infected Subjects who Failed Short-Duration All-Oral Therapy* in *AASLD*. 2015: San Francisco.

25. Emmanuel, B., et al., *Shortening the duration of therapy for chronic hepatitis C infection.* Lancet Gastroenterol Hepatol, 2017.

26. BMS. *Daclatasvir SPC*. 2017; Available from: https://<http://www.medicines.org.uk/emc/medicine/29129>.

27. Gilead Inc, *Sofosbuvir SPC.* 2017.

Appendix I - **Data Monitoring Committee Membership**

Data Monitoring Committee

Chair person: Professor Tim Peto (Oxford)

Independent members:

Professor John Dillon (Dundee)

Assoc. Prof. Dr. Pham Thi Le Hoa (University of Medicine and Pharmacy at Ho Chi Minh city)

appendix ii - Trial Assessment Schedule

Stratum A Initial Treatment

Stratum A Retreatment

Stratum B

Appendix III - Toxicity Gradings and Management

Common Toxicity Criteria for Adverse Events

<https://ctep.cancer.gov/protocolDevelopment/electronic_applications/docs/CTCAE_v5_Quick_Reference_8.5x11.pdf>

ULN = Upper Limit of Normal; LLN = Lower Limit of Normal

**General Instructions:**

If the need arises to grade a clinical adverse event (AE) that is not identified in the table, use the category “Estimating Severity Grade” located at the top of the table.

If the severity of an AE could fall under either one of two grades (e.g. the severity of an AE could be either Grade 2 or Grade 3) select the higher of the two grades for the AE.
